# Dynamic landscape mapping of humoral immunity to SARS-CoV-2 identifies non-structural protein antibodies associated with the survival of critical COVID-19 patients

**DOI:** 10.1038/s41392-021-00718-w

**Published:** 2021-08-17

**Authors:** Linlin Cheng, Xiaomei Zhang, Yu Chen, Dan Wang, Dong Zhang, Songxin Yan, Hongye Wang, Meng Xiao, Te Liang, Haolong Li, Meng Xu, Xin Hou, jiayu Dai, Xian Wu, Mingyuan Li, Minya Lu, Dong Wu, Ran Tian, Jing Zhao, Yan Zhang, Wei Cao, Jinglan Wang, Xiaowei Yan, Xiang Zhou, Zhengyin Liu, Yingchun Xu, Fuchu He, Yongzhe Li, Xiaobo Yu, Shuyang Zhang

**Affiliations:** 1 Department of Clinical Laboratory, State Key Laboratory of Complex Severe and Rare Diseases, Peking Union Medical College Hospital, Chinese Academy of Medical Science and Peking Union Medical College, Beijing, China; 2 State Key Laboratory of Proteomics, Beijing Proteome Research Center, National Center for Protein Sciences-Beijing (PHOENIX Center), Beijing Institute of Lifeomics, Beijing, China

**Keywords:** Infectious diseases, Infectious diseases

## Abstract

A comprehensive analysis of the humoral immune response to the severe acute respiratory syndrome coronavirus 2 (SARS-CoV-2) is essential in understanding COVID-19 pathogenesis and developing antibody-based diagnostics and therapy. In this work, we performed a longitudinal analysis of antibody responses to SARS-CoV-2 proteins in 104 serum samples from 49 critical COVID-19 patients using a peptide-based SARS-CoV-2 proteome microarray. Our data show that the binding epitopes of IgM and IgG antibodies differ across SARS-CoV-2 proteins and even within the same protein. Moreover, most IgM and IgG epitopes are located within nonstructural proteins (nsps), which are critical in inactivating the host’s innate immune response and enabling SARS-CoV-2 replication, transcription, and polyprotein processing. IgM antibodies are associated with a good prognosis and target nsp3 and nsp5 proteases, whereas IgG antibodies are associated with high mortality and target structural proteins (Nucleocapsid, Spike, ORF3a). The epitopes targeted by antibodies in patients with a high mortality rate were further validated using an independent serum cohort (*n* = 56) and using global correlation mapping analysis with the clinical variables that are associated with COVID-19 severity. Our data provide fundamental insight into humoral immunity during SARS-CoV-2 infection. SARS-CoV-2 immunogenic epitopes identified in this work could also help direct antibody-based COVID-19 treatment and triage patients.

## Introduction

The coronavirus disease 2019 (COVID-19) was declared a pandemic on 11 March 2020, by the World Health Organization (WHO).^1–3^ By 21 June 2021, a total of 178,423,323 cases of infection and 3,864,419 deaths were reported. COVID-19 is characterized by symptoms of viral pneumonia, such as fever, fatigue, dry cough, and lymphopenia. According to a report by the Chinese Center for Disease Control and Prevention that followed 72,314 COVID-19 patients, symptoms were mild, moderate, severe, or critical for 40%, 40%, 15%, or 5% of cases, respectively. Patients classified as “critical” have the most severe symptoms, with complications that include respiratory failure, shock, and/or multiorgan failure. Moreover, the mortality rate of critical patients is as high as 49%.^4^ Thus, identifying predictive biomarkers of survival in critical patients would be invaluable in providing the proper treatment for this group of patients.

COVID-19 is caused by infection with the severe acute respiratory syndrome coronavirus 2 (SARS-CoV-2). Antibodies to SARS-CoV-2 proteins, which are generated within a week of exposure, are important in identifying prior viral infection, evaluating humoral immunity, and performing epidemiological and vaccine studies.^5–8^ The SARS-CoV-2 genome encodes ten proteins: an ORF1ab polyprotein, four structural proteins (envelope, E; membrane, M; nucleocapsid, N; spike, S) and five accessory proteins (ORF3a, ORF6, ORF7a, ORF8, ORF10) (Supplementary Text).^9–11^ Notably, the ORF1ab polyprotein is cleaved into 15 or 16 non-structural proteins (nsps).^11^ The S protein plays a critical role in viral entry and, as such, is the major target for developing vaccines and neutralizing antibodies.^12^ Like the S protein, the nsps play important roles in viral transcription, replication, and assembly,^13–16^ and can elucidate immunological responses.^8,17,18^ Previous studies suggest that the level of SARS-CoV-2 antibodies is associated with COVID-19 severity, where most humoral antibodies target structural (N, S) and accessory (ORF3b, ORF8) proteins.^7,8,11,17,19^ However, a proteome-wide analysis of humoral immunological responses to the SARS-CoV-2 proteome in critical COVID-19 patients has not yet been performed.

In this work, we performed a longitudinal analysis of antibodies that are produced in response to SARS-CoV-2 infection in 104 serum samples of 49 critical COVID-19 patients across 1–83 days post symptom onset. The spatial distribution of antibody epitopes on SARS-CoV-2 proteins was thoroughly analyzed by epitope mapping and structural analyses. Furthermore, the antibodies associated with the survival of critical COVID-19 patients were identified and validated in an independent group of serum samples.

## Results

### SARS-CoV-2 antibody epitope detection with a peptide-based microarray

**Fig. 1:**
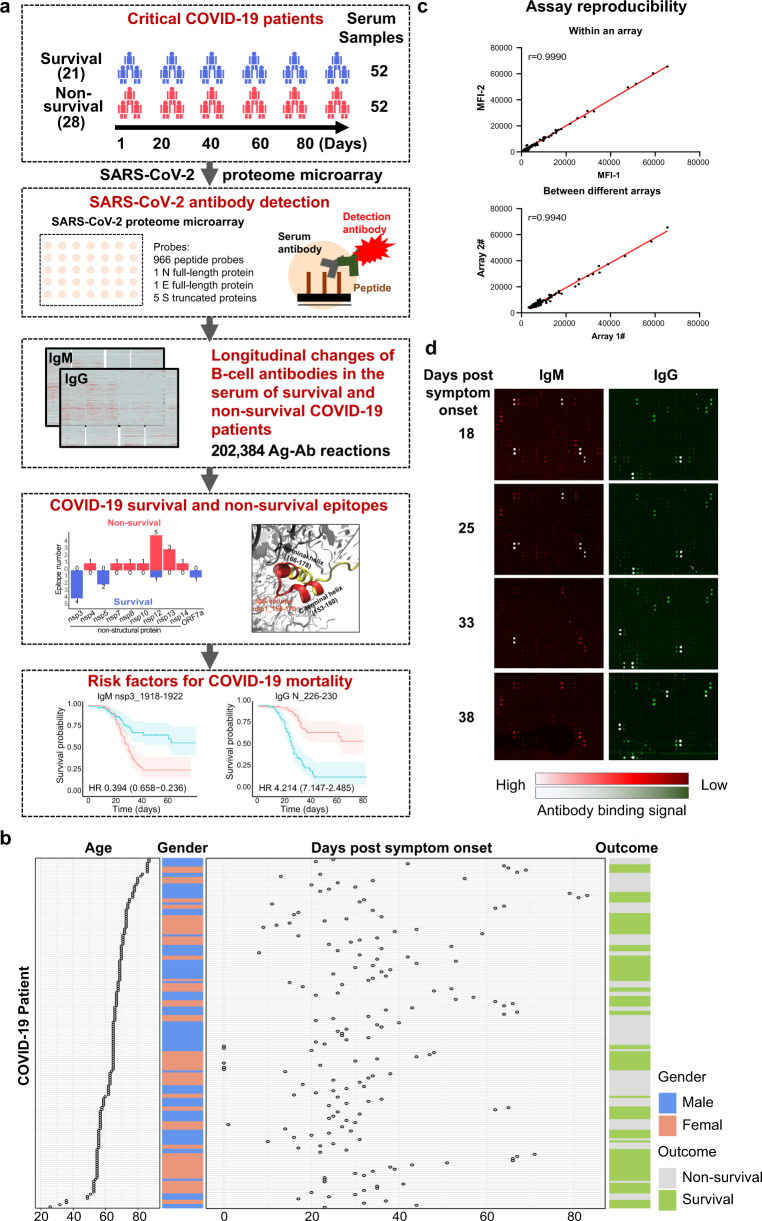
Detection of SARS-CoV-2 serum antibodies in critical COVID-19 patients. **a** Workflow of the longitudinal analyses of SARS-CoV-2 proteome antibodies in the serum of critical COVID-19 patients. **b** Characteristics of the COVID-19 patients in this study. **c** The reproducibility of serological antibody detection using the SARS-CoV-2 proteome microarray within an experiment and between different experiments. The r correlation was calculated within an array and between different arrays. **d** Representative fluorescent image of antibody detection using serum from a COVID-19 patient who did not survive at different days post symptom onset

A schematic illustration of our longitudinal analysis of SARS-CoV-2 proteome antibodies in critical COVID-19 patients is shown in Fig. 1a. All critical COVID-19 patients were diagnosed according to the Diagnosis and Management Plan of Pneumonia with New Coronavirus Infection (trial version 7) (National Health Commission & State Administration of Traditional Chinese Medicine; 3 March 2020) (Fig. 1b, Supplementary Tables 1 and 2). The SARS-COV-2 proteome microarray was printed using a tiled library of 966 peptides representing the wild-type sequence (Wuhan-Hu-1, GenBank: MN908947.3), in which each peptide was 15 amino acids long with a 5 amino acid overlap (Supplementary Table 3). Full-length recombinant N and E proteins and five S truncated recombinant proteins were also printed.^11^ All spots were printed in duplicate. After patient serological IgM and IgG antibodies bound to the peptide or protein spots on the microarray, fluorescently conjugated anti-human IgM or IgG detection antibodies were added. The fluorescent signal, proportional to the amount of patient antibody bound to the array, was measured using a compatible laser scanner (Supplementary Fig. 1).

The SARS-CoV-2 proteome microarray detects antibodies with high reproducibility, with intra- and inter-array correlations of 0.99 and 0.99, respectively (Fig. 1c). Antibody binding in a clinical cohort of 104 serum samples from 49 critical COVID-19 patients was assessed with the SARS-CoV-2 proteome microarray (Supplementary Table 1). Representative array images of antibody detection using serum from a critical COVID-19 patient are shown in Fig. 1d. As expected, the number of IgM antibody signals decreased while the number of IgG antibody signals increased between 18 and 38 days post symptom onset. These results support the use of our microarray to detect SARS-CoV-2 antibodies in COVID-19 patients.

### Dynamic landscapes of humoral immunological responses to SARS-CoV-2 proteins

**Fig. 2:**
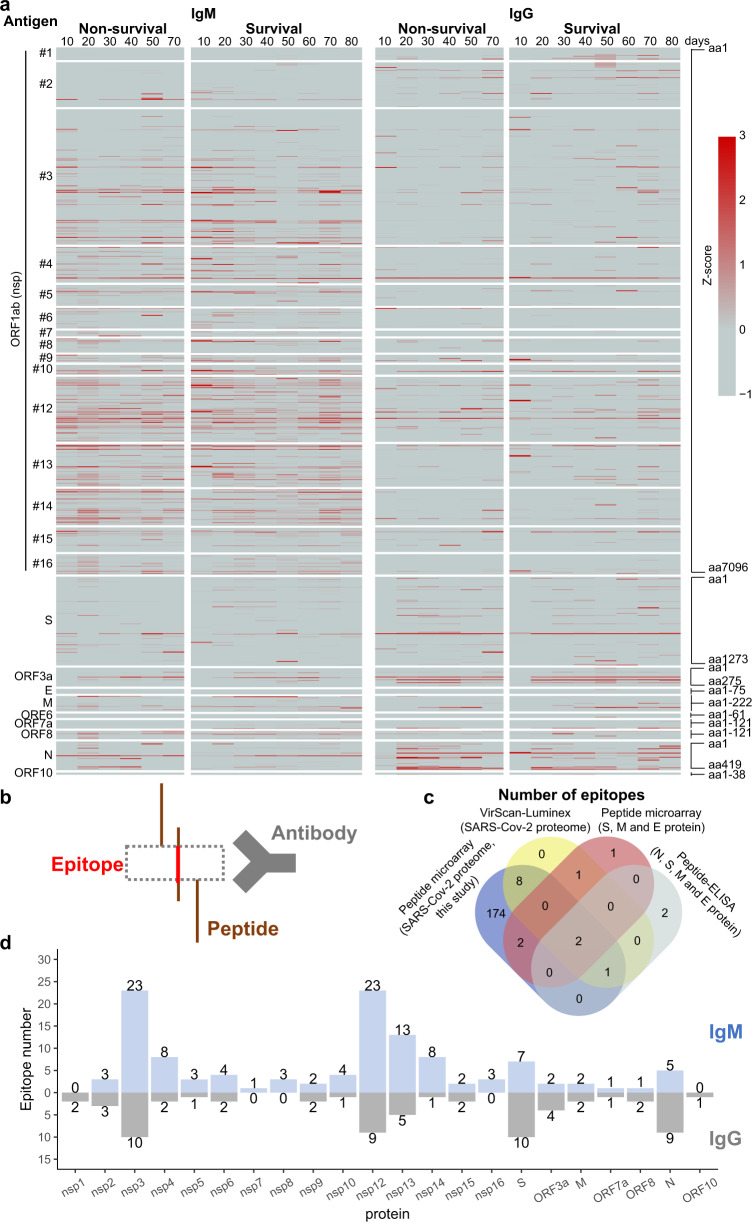
Longitudinal changes of SARS-CoV-2 proteome antibodies in critical COVID-19 patients. **a** Longitudinal detection of SARS-CoV-2 IgM and IgG antibodies in critical COVID-19 patients. The raw data for each peptide and protein on the array were normalized to the z-score. The left *y*-axis represents the different SARS-CoV-2 proteins. The right y-axis represents the amino acids of each protein sequence, from the N-terminus (left) to C-terminus (right). The top *x*-axis shows the number of days post symptom onset in 10-day intervals. **b** Schematic illustration of epitope identification via epitope mapping. **c** Validation of reported peptide epitopes with data from this study. **d** Number of IgM and IgG peptide epitopes identified within each SARS-CoV-2 protein

The longitudinal changes of antibody responses to the SARS-CoV-2 proteome in critical COVID-19 patients 1–83 days post symptom onset are illustrated in Fig. 2a. IgM and IgG antibodies were generated to most SARS-CoV-2 proteins throughout this time span. While IgM antibodies preferentially bound to ORF1ab-derived nsps, the IgG antibodies primarily targeted N, S, and two accessory proteins, ORF3a and ORF8 (Fig. 2a).

One hundred and eighteen (118) IgM and sixty-nine (69) IgG antigenic epitopes with a z-score higher than 1.96 (95% confidence interval) in at least three serum samples were identified as “hits” using sequence alignment as previously described (Fig. 2b).^11,20^ We then confirmed that 91.7% (11/12) and 60% (3/5) of these epitopes were associated with disease severity using VirScan and peptide-based ELISA, respectively (Fig. 2c).^17,21^ Moreover, 66.7% (4/6) of the immunodominant epitopes were previously identified in a COVID-19 convalescent population (Fig. 2c).^22^ The peptide epitopes identified in our study constitute the largest database of IgM and IgG antibody epitopes in critical COVID-19 patients to date (Fig. 2c, Supplementary Tables 4 and 5). These data, in conjunction with work from previous studies, indicate that antibodies produced in more severe COVID-19 cases target a larger breadth of binding epitopes than less severe cases (Supplementary Tables 4 and 5, Supplementary Figs. 2–3).^11,17,23–28^

**Fig. 3:**
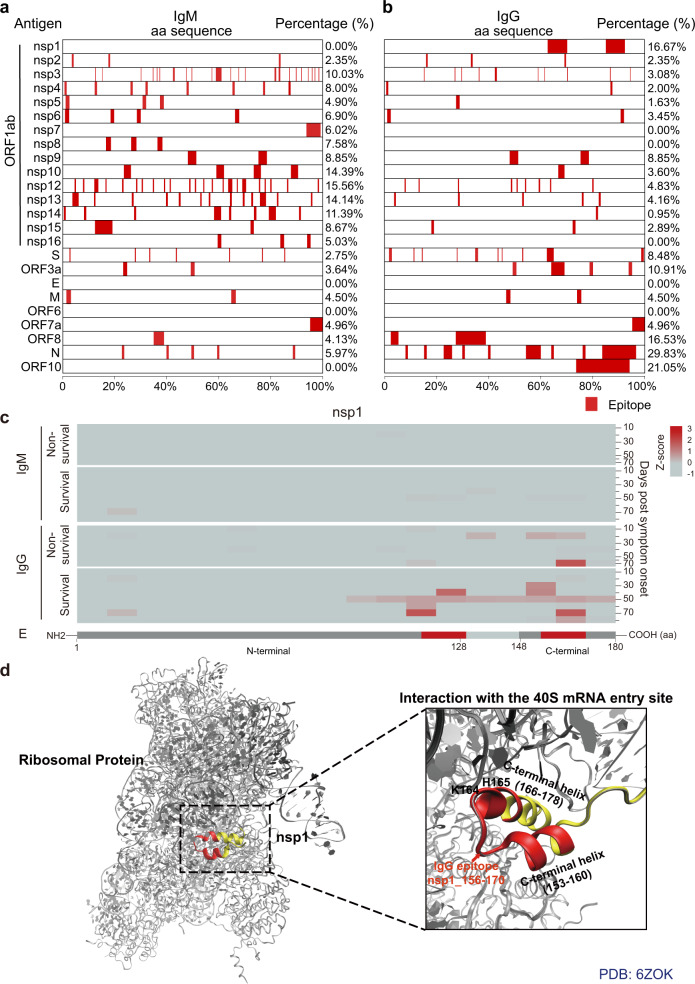
The landscape of antibody epitopes identified in critical COVID-19 patients. **a**, **b** Landscapes of IgM and IgG antibody epitopes identified within the SARS-CoV-2 proteome, respectively. The left *y*-axis is the protein name and the right y-axis is the percent coverage of peptide epitopes on each protein sequence. **c** Longitudinal changes of IgM and IgG antibody epitopes identified within the SARS-CoV-2 nsp1 protein. **d** Structural analyses of IgG antibody epitopes within the SARS-CoV-2 nsp1 protein. The epitopes within the functional domain that may interfere with protein activity are labeled in red

Among the proteins encoded by the SARS-CoV-2 genome, nsp3 and nsp12 had the largest number of IgM epitopes (23) (Fig. 2d, Supplementary Table 4) while the nsp3 and S proteins had the largest number of IgG epitopes (10) (Fig. 2d, Supplementary Table 5). We further defined the peptide-based immunogenicity (PBI) for all SARS-CoV-2 proteins by calculating the percentage (%) of the sum total length of the IgM or IgG epitopes relative to the full-length of the protein (Figs. 3a and b). The results show that nsp12 had the strongest IgM antibody PBI (15.56%) (Fig. 3a). The N protein had the strongest IgG antibody PBI (29.83%) (Fig. 3b). Notably, no IgM or IgG antibodies bound to ORF6- or E-related peptides in this study; as such, these proteins were assigned as non-immunogenic proteins.

### Structural analyses of antibody epitopes on SARS-CoV-2 nsps

The structural analyses of the antibody epitopes on the SARS-CoV-2 structural and accessory proteins are shown in Supplementary Figs. 4–7, 16–20, and Supplementary Table 6. The spatiotemporal resolution of antibody epitopes to S and N, two proteins that have been previously well-characterized, are shown in Supplementary Figs. 4–7. Notably, the locations of all antibody epitopes identified in this study do not overlap with amino acids that are known to be glycosylated (Supplementary Fig. 8).^29^ Since the peptide array identifies linear binding epitopes, our data indicate that linear epitopes may not be glycosylated in vivo.

**Fig. 4:**
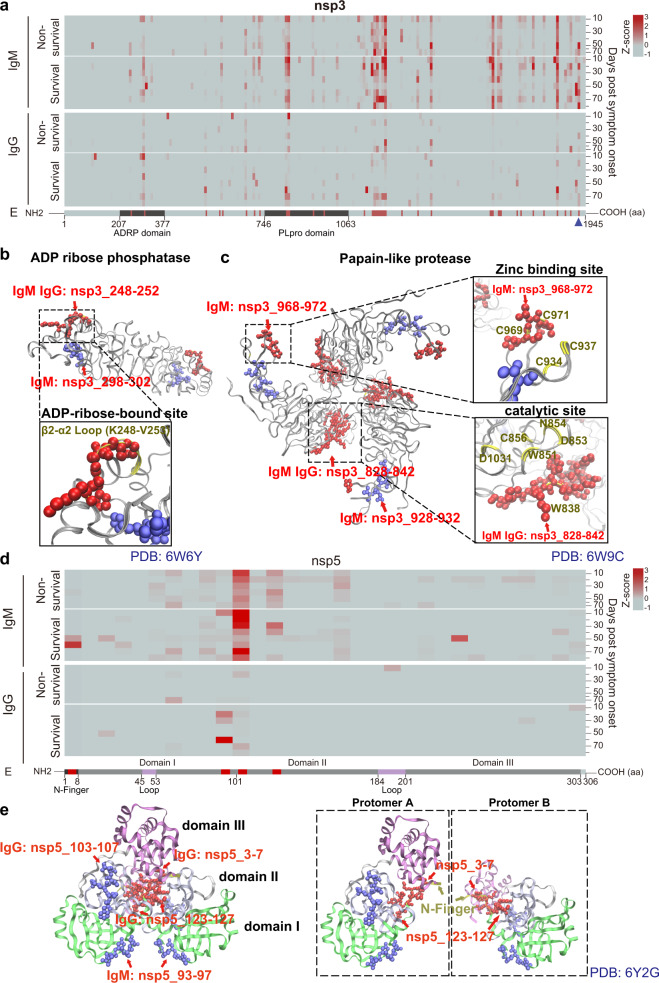
Dynamic changes of antibody epitopes within SARS-CoV-2 proteases. **a** Longitudinal changes of IgM and IgG antibody epitopes identified within the SARS-CoV-2 nsp3 protein. **b**, **c** Structural analyses of IgM and IgG epitopes within the ADP ribose phosphatase and papain-like protease, respectively. **d** Longitudinal changes of IgM and IgG antibody epitopes identified within the SARS-CoV-2 nsp5 protein. **e** Structural analyses of IgM and IgG epitopes within the nsp5 protein. The epitopes within the functional domain that may interfere the protein activity are labeled in red. ADRP ADP-ribose phosphatase domain, PLpro papain-like protease domain

**Fig. 5:**
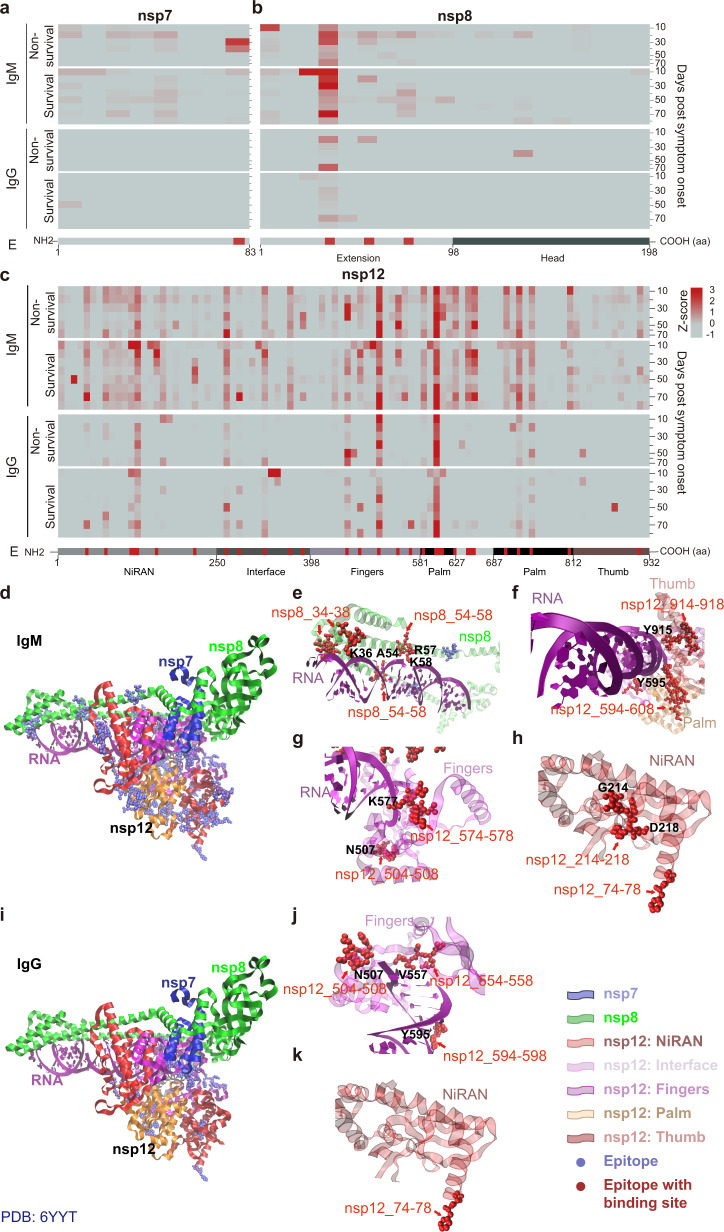
Dynamic changes of antibody epitopes within the SARS-CoV-2 RNA-dependent RNA polymerase (RdRp). **a**–**c** Longitudinal changes of IgM and IgG antibody epitopes identified within the SARS-CoV-2 nsp7, 8, and 12 proteins, respectively. **d**–**h** Structural analyses of IgM epitopes on the RdRp complex, nsp8-RNA interaction interface, Thumb and Palm domain, Fingers domain, and N-terminal nidovirus RdRp-associated nucleotidyltransferase (NiRAN) domain, respectively. **i**–**k** Structural analyses of IgG epitopes on the RdRp complex, nsp8-RNA interaction interface, Fingers domain and NiRAN domain, respectively. The epitopes within the functional domain that may interfere the protein activity are labeled in red

**Fig. 6:**
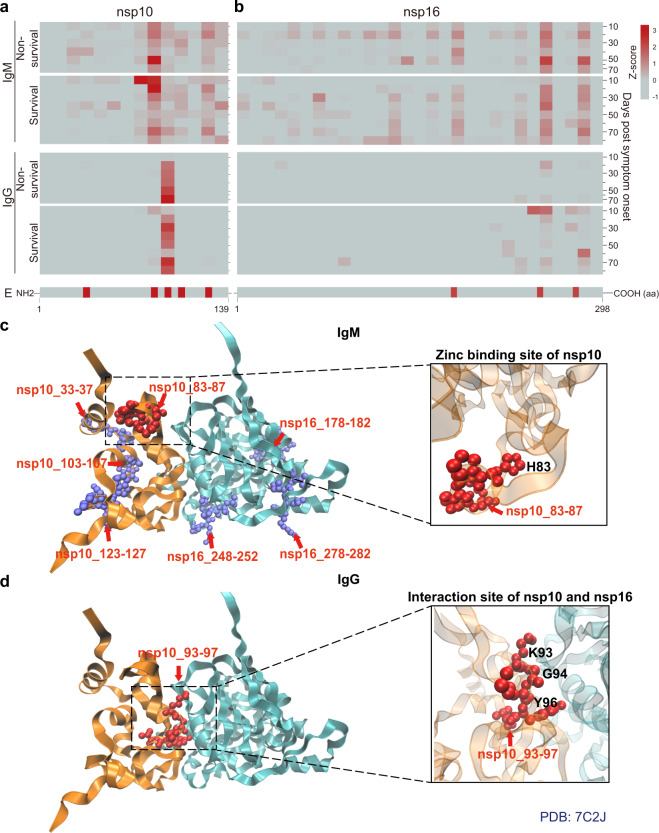
Dynamic changes of antibody epitopes within the SARS-CoV-2 nsp10/nsp16 2′-O-methylase complex. **a**, **b** Longitudinal changes of IgM and IgG antibody epitopes identified within the nsp10/nsp16 2′-O-methylase complex, respectively. **c**, **d** Structural analyses of IgM and IgG epitopes within the nsp10/nsp16 2′-O-methylase complex. The epitopes within the functional domain that may interfere the protein activity are labeled in red

The SARS-CoV-2 nsps are essential to viral invasion, transcription, and replication.^14,15,30^ The systematic profiling of antibody epitopes within SARS-CoV-2 nsps proteins would help understand the adaptive immune response to COVID-19 and may help identify targets for generating antibodies to treat COVID-19 (Figs. 3–6, Supplementary Figs. 9–15).^31^ Nsp1 is a major virulence factor that inhibits the translation of host messenger RNA (mRNA) by binding to the ribosomal mRNA channel through its C-terminus.^32,33^ Our data identified two IgG epitopes within nsp1 (nsp1_116-130, nsp1_156-170) (Fig. 3c, Supplementary Table 5). One of these epitopes (nsp1_156-170) at the C-terminus contains residues K164 and H165, which are necessary for the nsp1 binding to the ribosome. The epitope also is located within two C-terminal helices (residues 153–160 and 166–178) that interact with proteins involved translation initiation (i.e., uS5, Us3, h18).^32,34^ Antibody binding of this epitope may therefore enable host protein translation (Fig. 3d).^33,34^

Nsp3 is a large, multidomain protein that includes an ADP-ribose phosphatase domain (ADRP) and a papain-like protease domain (PLpro).^35,36^ The ADRP domain binds ADP ribose and interferes with the host immune response by removing ADP ribose from ADP-ribosylated proteins or RNA.^37^ The PLpro is a protease that is essential in producing a functional replicase complex that enables viral replication. In this work, 23 IgM epitopes and 10 IgG epitopes to nsp3 were identified (Figs. 2d, 4a, Supplementary Tables 4 and 5). An epitope targeted by both IgM and IgG antibodies (nsp3_248-252) is located within the β2–α2 (K248-V253) loops of the ADRP domain that binds ADP ribose (Fig. 4b). The IgM and IgG epitope (nsp3_828-842) that contains a catalytically important residue, W838, is located within the PLpro domain while the epitope (nsp3_968-972) is located within the Zinc binding site of PLpro and contains two of the four important zinc finger residues, C969 and C971 (Fig. 4c).

The nsp5 protein is also known as the 3C-like protease (3CLpro), which mediates the cleavage and subsequent activation of polyproteins involved in viral replication.^38^ As such, it has become a target-of-interest for COVID-19 therapy.^39^ We identified three IgM epitopes (nsp5_3-7, nsp5_103-107, nsp5_123-127) and one IgG epitope (nsp5_93-97) to nsp5 (Fig. 4d, Supplementary Tables 4 and 5). One epitope (nsp5_3-7) is located within the N-terminal seven residues (“N finger”), which is necessary for nsp5 homodimerization.^39^ Importantly, the dimer is the catalytically active form of nsp5, while the monomer is mostly inactive.^39^ Two epitopes (nsp5_3-7, nsp5_123-127) are located at the dimerization interface of protomer A and B (Fig. 4e). Antibodies that target these sites may inhibit nsp5 activation. Finally, two epitopes (nsp5_93-97, nsp5_103-107) are located within domain I and domain II, respectively (Fig. 4e).

The SARS-CoV-2 RNA-dependent RNA polymerase (RdRp) is comprised of nsp12 and two accessory subunits, nsp7 and nsp8, which are used for the replication of the viral genome and transcription of viral genes.^15^ In this study, nsp12 had the maximal number of IgM epitopes, followed by nsp8 and nsp7 (Fig. 5a–c). The majority of the identified epitopes are distributed on the surface of the RdRp complex (Fig. 5d and i). Although two IgM epitopes (nsp8_34-38, nsp8_54-58) of nsp8 are located on the outside of the RdRp complex, four of the residues (K36, A54, R57, K58) interact with viral RNA (Fig. 5e).^15^ Two IgM epitopes (nsp12_594-608, nsp12_914-918) to the nsp12 protein are located within the Palm and Thumb domains while two IgM epitopes (nsp12_504-508, nsp12_574-578) are located within the Fingers domain (Fig. 5f, g). All of these epitopes come into contact with viral RNA. Two other IgM epitopes (nsp12_74-78, nsp12_214-218) are located within the N-terminal nidovirus RdRp-associated nucleotidyltransferase (NiRAN) domain that binds ADP-Mg^2 + ^through three residues: H75, G214, and D218 (Fig. 5h). As for IgG epitopes, three epitopes (nsp12_504-508, nsp12_554-558, nsp12_594-598) within the Fingers domain bind to RNA through the residues N507, V557, and Y595 (Fig. 5j).^15^ Similarly, one IgG epitope (nsp12_74-78) is located within the NiRAN domain, which binds ADP-Mg^2 + ^through the residue H75 (Fig. 5k).^15^

Nsp10 and nsp16 form a nsp10/nsp16 2′-O-methylase complex that catalyzes the methylation of the penultimate nucleotide of the viral RNA cap at the ribose 2′-O position. The process is used to mimic cellular mRNAs and prevent the recognition of viral RNAs by the host immune system.^40^ The immunogenicity of nsp10 and nsp16 was low in this study, with only 5 and 3 epitopes identified, respectively (Fig. 2d, Fig. 6a and b). However, structural analyses indicated that the IgM epitope (nsp10_83-87) is located within the zinc binding site (Fig. 6c) while the IgG epitope (nsp10_93-97) is located within the interaction site of nsp10 and nsp16 (Fig. 6d). The antibodies to these two epitopes might inhibit zinc binding, the stabilized conformation of nsp10, and the formation of the nsp10/nsp16 complex, thus enabling the recognition of viral RNA by the immune system.^41^

### SARS-CoV-2 antibodies are associated with the COVID-19 survival

**Fig. 7:**
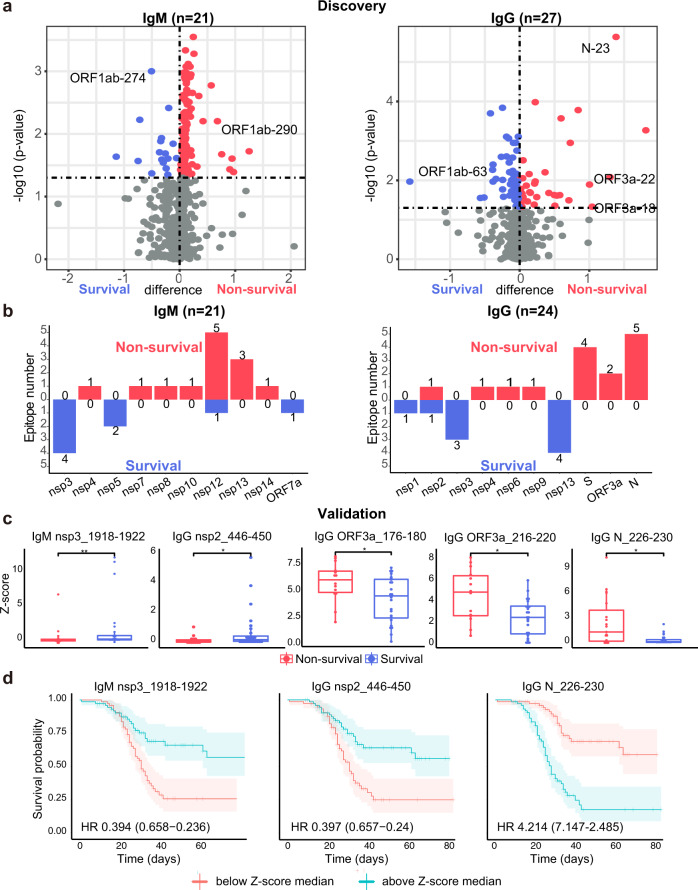
Association between SARS-CoV-2 antibodies and the survival of critical COVID-19 patients. **a**, **b** Identification of SARS-CoV-2 IgM and IgG antibodies associated with the survival and non-survival of critical COVID-19 patients, respectively. The antibody candidates were identified using a *t*-test with a *p*-value < 0.05 and an immunogenicity with a z-score > 1.96 in ≥3 serum samples. **b** IgM and IgG peptide epitopes within SARS-CoV-2 proteins associated with the survival and non-survival of COVID-19 patients, respectively. **c** Validation of epitopes associated with survival and non-survival in an independent serum cohort. **d** Kaplan–Meier curve analyses of SARS-CoV-2 antibodies as potential risk factors of COVID-19 mortality. The Kaplan–Meier curves show the survival probability of patients with high versus low antibody levels since symptom onset. Grouping criteria (cutpoints) are provided in the graphs. Hazard ratios (HR) for high versus low antibody levels are provided with p-values from log-rank tests

To identify the antibody epitopes associated with COVID-19 prognosis, we compared the epitopes between critical COVID-19 patients who survived and did not survive. Forty-eight (21 IgM, 27 IgG) immunogenic peptides were identified by epitope mapping with a p-value≦0.05 and z-score > 1.96 in ≥3 serum samples (Fig. 7a, Supplementary Table 7). Due to peptide overlap, 45 of these peptides were unique, including 21 IgM epitopes and 24 IgG epitopes (Fig. 7b). No peptide epitope was targeted by both IgM and IgG antibodies.

Notably, the IgM and IgG epitopes associated with survival are mainly within the nsp3 and nsp5 proteins (Fig. 7b), whereas the IgM and IgG epitopes associated with non-survival are within the RdRp and structural proteins (S, ORF3a, N), respectively (Fig. 7b).

To validate these biomarker candidates, we screened the serum from an independent “validation” cohort containing 56 serum samples from 11 COVID-19 patients who survived and 11 patients who did not (Supplementary Table 2). We then performed statistical analyses using the same criteria as described above. Five (5) peptide epitopes significantly associated with COVID-19 mortality in the discovery cohort were also observed in the validation cohort (*p* value ≤ 0.05; z-score >1.96 in ≥3 serum samples), including IgM antibodies to nsp3_1918-1922 and IgG antibodies to nsp2_446-450, ORF3a_176-180, ORF3a_216-220, and N_226-230 (Fig. 7c).

We further performed the Kaplan-Meier survival analyses of SARS-CoV-2 antibodies, and identified 3 SARS-CoV-2 antibody epitopes as potential risk factors to COVID-19 survival (Fig. 7d). These antibody epitopes are IgG-N_226-230 (HR 4.214, 95% CI 7.147–2.485, *p* < 0.001), IgM-nsp3_1918-1922 (HR 0.394, 95% CI 0.658–0.236, *p* < 0.001), and IgG-nsp2_446-450 (HR 0.397, 95% CI0.657–0.24, *p* < 0.001) (Supplementary Table 7, Supplementary text). Notably, the increase of antibodies to two epitopes on nsps proteins (nsp3_1918-1922, nsp2_446-450) indicates longer survival, whereas the increase of antibodies to an N protein epitope (N_226-230) is associated with a poor prognosis (Fig. 7d). All these results demonstrate that SARS-CoV-2 antibodies may serve as potential risk factors of COVID-19 mortality and should be investigated in a larger independent cohort in the future.

### Global correlation mapping of SARS-CoV-2 antibodies and clinical variables

**Fig. 8:**
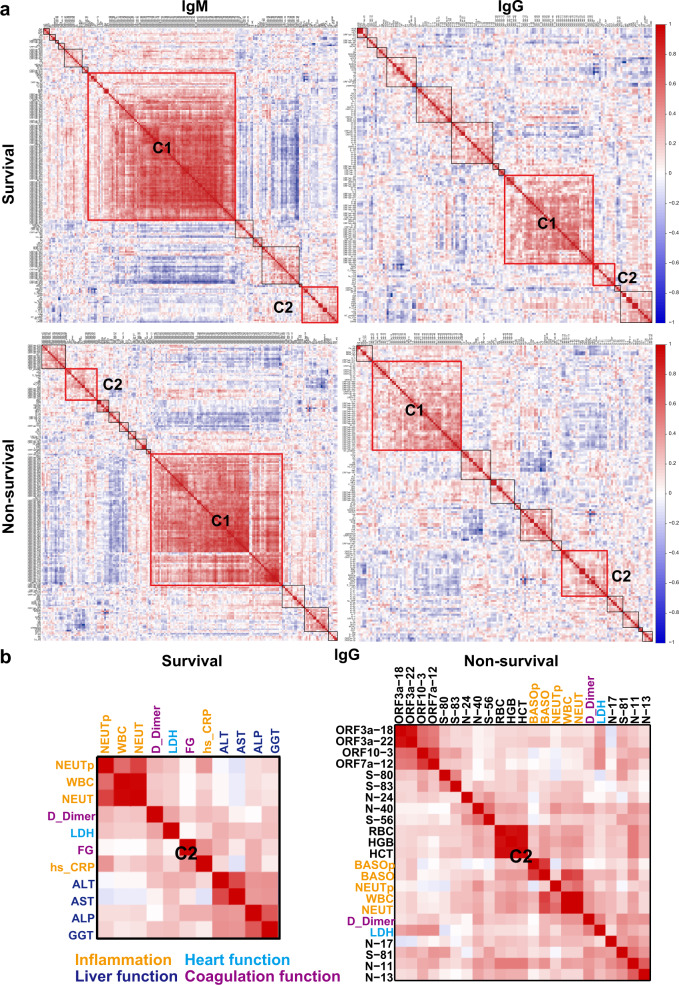
Correlations between antibodies to structural proteins and clinical variables associated with COVID-19 survival. **a** Global correlation map of the SARS-CoV-2 proteome IgG antibodies and clinical variables in the survival and non-survival COVID-19 patient groups. **b** Differential correlations of the clinical variables and SARS-CoV-2 IgG antibodies between survival and non-survival COVID-19 groups in the cluster #2 group. The rainbow color from blue to red corresponds to the correlation of two variables from −1 (low correlation; blue) to +1 (high correlation; red)

A global correlation analysis was performed to determine whether clinical variables are associated with SARS-CoV-2 antibodies in critical COVID-19 patients (Supplementary Tables 8 and 9). After correlating all variables with each other and performing hierarchical clustering, four global correlation maps were constructed containing 39,204 (survival, IgM); 39,204 (non-survival, IgM); 22,500 (survival, IgG); and 22,500 (non-survival, IgG) Pearson correlation coefficients (Fig. 8a and Supplementary Figs. 21–24).

Overall, the IgM and IgG global correlation map profiles were different. However, the largest cluster (C1) in both maps include antibodies to ORF1ab (Supplementary Fig. 25). The clinical variables that clustered together as the C2 cluster of the IgM-survival group correlation map include coagulation [D-dimer, active partial thromboplastin time (APTT), prothrombin time (PT), international normalized ratio (INR), mean platelet volume (MPV), platelet-large cell ratio (P_LCR)], liver and kidney function [lactate dehydrogenase (LDH), indirect bilirubin (IBIL), total bilirubin (TBIL), direct bilirubin (DBIL), creatinine (CR), uric acid (UA), Urea, N-terminal pro b-type natriuretic peptide (NT-proBNP)], heart function [cTn1, myoglobin (Mb), creatine kinase-MB (CKMB)], and inflammation [neutrophil (NEUT), the percentage of neutrophils (NEUTp), C-reactive protein (CRP) and white blood cell (WBC)]. The different clinical variables observed in our study may reflect the involvement of multiple organs and systems during COVID-19 infection of critical COVID-19 patients.^42^ However, the inflammation variables (NEUT, NEUTp, CRP) separated from the C2 cluster in the non-survival group. The remaining variables (coagulation, liver and kidney function, heart function) clustered with IgM antibodies to seven ORF1ab peptides (Supplementary Fig. 26).

The IgG correlation map representing data from patients who survived COVID-19 also showed that some coagulation variables [D-dimer, fibrinogen (FG)] clustered with liver function [LDH, alanine aminotransferase (ALT), aspartate aminotransferase (AST), alkaline phosphatase (ALP), gamma-glutamyl transferase (GGT)] and inflammation [high-sensitivity C-reactive protein (hs_CRP), WBC, NEUTp, NEUT]. In the non-survival group, however, the clinical variables of coagulation (D-dimer) and inflammation [(WBC, NEUT, NEUTp, basophil (BASO), percentage of basophils (BASOp)] associated with higher COVID-19 mortality exclusively clustered with IgG antibodies targeting peptides from structural (N, S) and accessory (ORF3a, ORF7a, ORF10) proteins (Fig. 8b). These results further demonstrate the association between antibody epitopes within structural proteins and COVID-19 patients with a high mortality rate (Fig. 7).

## Discussion

Previous studies have indicated that the immune responses in critical COVID-19 patients might differ from patients classified as asymptomatic, mild, moderate, or severe (Supplementary Tables 4 and 5, Supplementary Figs. 2–3).^11,17,23–28^ In this work, we comprehensively analyzed the humoral immune response to SARS-CoV-2 in critical COVID-19 patients for the first time at amino acid resolution using a SARS-CoV-2 proteome microarray (Fig. 1a). We further determined the association between antibody binding epitopes and COVID-19 survival.

Our data show that the humoral response to the SARS-CoV-2 proteome is dynamic (Fig. 2) and target unique epitopes (Fig. 3a). The nsps generated from the ORF1ab polyprotein have the largest number of epitopes that are targeted by both IgM (84.7%, 100/118) and IgG (58.0%, 40/69) antibodies (Fig. 2d). While the IgM antibodies targeted more epitopes to nsps than IgG antibodies, the IgG antibodies targeted epitopes within the structural (N, S) and accessory (ORF3a, ORF8) proteins (Figs. 2a, d, 3a, b). Furthermore, the IgM- and IgG-specific epitopes were different, demonstrating the different mechanisms used for IgM and IgG antibody production (Supplementary Fig. 6a, red arrow).^43^ Notably, we previously identified three IgM epitopes (S_816-820, ORF3a_136-140, N_206-210) and one IgG epitope (S_816-820) as potential early diagnostic biomarkers of COVID-19 infection.^11^

Structural analyses indicated that most immunogenic epitopes tend to be located on the surface of SARS-CoV-2 proteins (Figs. 3–6, Supplementary Figs. 4–7, 12–13, 15, 17, 19). These data make sense since amino acids at the surface of SARS-CoV-2 proteins are more exposed for detection by the adaptive immune response. Many of the IgM and IgG epitopes identified in this current study include residues that are critical for nsps to maintain their functions (Figs. 3d, 4–6). Thus, the host immune response may defend against SARS-CoV-2 infection by generating antibodies that inhibit viral invasion (S), RNA protection (nsp10/nsp16 2′-O-methylase complex), replication and transcription (RdRp), polyprotein processing (nsp3 PLpro domain, nsp5 proteases) while also helping to activate the host’s innate immunity (nsp1, nsp3 ADRP domain) (Figs. 3–6, Supplementary Figs. 4–5).^31,44,45^ The hypothesis can be supported by the nanobody that targets the nsp9 of the porcine reproductive and respiratory syndrome virus (PRRSV), which showed antiviral activity by inhibiting viral genome replication and transcription.^46,47^

Nanobodies, which are comprised of the antibody binding fragment of the heavy chain (VHH), have been recently investigated as diagnostic and therapeutic tools for cancer, autoimmune, psoriasis and infectious diseases.^48–53^ Their advantages include high affinity and specificity, small size, thermostability, ability to penetrate deep tissue, they are easy to engineer.^54–57^ Several nanobody candidates have entered clinical trials, including an anti-HER2 nanobody for detection of HER2 expression in breast cancer ([^131^I]-SGMIB antiHER2 VHH1),^51^ a nanobody targeting IL-6 in rheumatoid arthritis (ALX-0061),^58^ and a trivalent nanobody that neutralizes respiratory syncytial virus lower respiratory tract infection (ALX-0171).^59^ The nanobody, caplacizumab, treats acquired thrombotic thrombocytopenic purpura,^52^ and was the first nanobody to be approved by the U.S. Food and Drug Administration (FDA) in 2019. The use of nanobodies to fight against the spread of SARS-CoV-2 has also been explored. Two specific nanobodies, Nb91-hFc and Nb3-hFc, demonstrated antiviral activity by neutralizing SARS-CoV-2 pseudoviruses in vitro.^60^ Another nanobody produced by a naïve llama single-domain antibody library and PCR-based maturation showed neutralizing activity against live SARS-CoV-2.^61^ Our data may help direct the development of nanobodies targeting nsps for COVID-19 therapy.^31,44,45,62^

SARS-CoV-2 antibodies have been previously associated with COVID-19 severity.^5,7,63^ In this work, we identified 45 immunogenic epitopes indicative of COVID-19 prognosis. Antibodies associated with increased survival targeted nsp3 and nsp5 proteases, whereas IgG antibodies associated with increased mortality targeted structural proteins (N, S, ORF3a) (Figs. 7b, 8). Furthermore, two potential nsps antibody biomarkers (IgM, nsp3_1918-1922; IgG, nsp2_446-450) of critical COVID-19 patient prognosis were validated in an independent patient cohort (Fig. 7). While it is still unclear why antibodies to nsps could be associated with the survival of COVID-19 patients, the nsps are involved in critical steps of viral infection, including SARS-CoV-2 RNA protection, replication, and transcription, polyprotein procession, and in-activation of the host innate immunity. Thus, the generation of antibodies to nsps may be the immune system’s way to defend against COVID-19. Further investigation is warranted. Altogether, these results indicate that the immune response may use different approaches to fight SARS-CoV-2 infection.

There are several limitations in this study. First, the number of clinical serum samples in this study is limited, and the results should be validated in a large different cohort in the future. Second, some native SARS-CoV-2 epitopes identified by the immune system may not be detected with our peptide-based array, such as conformational or post-translational modifications.^17,29,64^ Finally, some antibody epitopes detected by other methods, such as VirScan, were not identified in this work. Possible reasons for this discrepancy may include the fact different clinical cohorts, technologies, assay parameters, and selection thresholds were employed.^17,23–28^

## Conclusion

We mapped the dynamic epitope landscape of humoral antibodies to the SARS-CoV-2 proteome in critical COVID-19 patients. Our data revealed IgM and IgG antibody signatures that are associated with patient survival. Altogether, our data provide fundamental insights into the longitudinal humoral immune response to SARS-CoV-2 infection and a valuable resource to the COVID-19 scientific community.

## Materials and methods

### Clinical sample

Seventy-one (71) COVID-19 patients classified as “critical” were included in this study between February and April 2020. All patients were recruited from an intensive care unit (ICU) in the Sino-French New City Branch of Tongji Hospital in Wuhan, China, which was managed by a multidisciplinary team from Peking Union Medical College Hospital (PUMCH).

COVID-19 patient diagnosis and classification were determined according to the Chinese Recommendations for Diagnosis and Treatment of Novel Coronavirus Infection (trial version 7).^65^ Patients classified as critical cases had any of the following features at the time of, or after, admission: (1) respiratory failure and require mechanical ventilation; (2) shock incidence; or (3) admission to ICU with other organ failure. All critical patients were divided into either the non-survival group or the survival group. The demographic features, comorbidities, symptoms and signs, laboratory information, treatment, and outcome of the patients were collected from electronic medical records at the time of admission (Supplementary Tables 1 and 2). All serum samples were centrifuged at 12,000 rpm for 10 min at 4 °C. The study was approved by the Research Ethics Commission of PUMCH (ZS-2303) and the requirement for informed consent was waived by the Ethics Commission.

### Preparation of the SARS-CoV-2 proteome microarray

The SARS-CoV-2 proteome microarray containing 966 tiled peptides, full-length N, full-length E, and truncated S proteins were prepared as previously described.^11^ Briefly, the peptide sequences were derived from the SARS-CoV-2 isolate Wuhan-Hu-1 (GenBank: MN908947.3), in which each peptide was 15 amino acids long with a 5 amino acid overlap. All peptides were labeled at the C-terminus with biotin and synthesized by either Guoping Pharmaceutical Company (Anhui, China) or China Peptides (Shanghai, China). All SARS-CoV-2 N, E, and S proteins were purchased from Sino Biological, Inc. (Beijing, China). The peptides and proteins were printed onto a three-dimensional (3D) modified slide surface (Capital Biochip Corp, Beijing, China) in parallel and in duplicate. Negative controls included phosphate-buffered saline (PBS), bovine serum albumin (BSA, 100 μg/mL) (Sigma-Aldrich, MO, USA), and hemagglutinin (HA) peptides (500 μg/mL) (China Peptides, Shanghai, China). Positive controls included biotinylated BSA (100 μg/mL), human IgG and IgM (10 μg/mL), and Polio peptides (500 μg/mL) (China Peptides, Shanghai, China). The peptide microarrays were stored at −20 °C until ready to use.

### Detection of serological antibodies in COVID-19 patients using a SARS-CoV-2 proteome microarray

The SARS-CoV-2 proteome array described above was assembled in an incubation tray and then blocked with 5% (w/v) milk in 1× PBS with 0.05% (v/v) Tween-20 (T) for 10 min at room temperature. After washing with PBST three times, the resulting array was incubated with 100-fold diluted serum in 5% (w/v) milk in PBST for 30 min at room temperature with gentle shaking. After washing again, the array was then incubated for 30 min with a mixture containing Cy3 Affinipure donkey anti-human IgG (H + L) and Alexa fluor 647 Affinipure goat anti-human IgM FC5µ antibody (Jackson ImmunoResearch, USA) (2 μg/mL). Finally, the array was washed with PBST buffer, dissembled from the tray, and dried via centrifugation for 2 min at 2000 rpm. The array was scanned with a GenePix 4300 A microarray scanner (Molecular Devices, Sunnyvale, CA, USA), and signals extracted using GenePix Pro7 software (Molecular Devices, Sunnyvale, CA, USA). These experiments were performed in a biosafety level 3 (BSL3) laboratory.

### Structural analyses of immunogenic epitopes

The Protein Data Bank (PDB) files were first downloaded from the structure databases of NCBI (https://www.ncbi.nlm.nih.gov/structure/?term=). The software VMD 1.9.4 was then used to perform the 3D structural analyses of immunogenic epitopes of the viral proteins. Different proteins in the complex or different domains within one protein were annotated with different “ColorID” and with the drawing method of “Ribbons”. The immunogenic epitopes were annotated by coloring method of “ColorID” and the drawing method of “CPK” style.

### Statistical analysis

The median of continuous variables (interquartile ranges, IQR) were compared with the *t*-test or Mann–Whitney *U* test; categorical variables were expressed as a percentage and compared by a Chi-squared test or Fisher’s exact test between non-survivors and survivors (Supplementary Tables 1 and 2). R version 3.5.2 was used to perform the statistical analyses.

The raw data for each peptide and protein on the array were normalized to the z-score. The reactive peptides were defined as those with *p*-value < 0.05 and a z-score >1.96 in ≥3 serum samples. For the differential analysis of each peptide or protein, the difference of the mean z-score between the survival and non-survival groups was determined with a *t*-test. A *p*-value < 0.05 was considered as statistically significant. Epitopes with a z-score difference between survival and non-survival groups >0 were considered to be related to non-survival, while z-score difference <0 were considered to be related to survival.

The survival analysis was implemented with the R package survminer. The hierarchical clustering analysis was implemented and plotted with the R package pheatmap. The correlation analysis for the immunogenic peptides and the clinic indicators was implemented and plotted with the R package corrplot.

## Supplementary information

Supplementary Material

Supplementary Table 3

Supplementary Table 7

Supplementary Table 9

## Data Availability

These data are available in the iProX database (www.iprox.org, accession number IPX0003181000). The files updated contain the sample ID, the survival and non-survival groups of critical COVID-19 patients, and the normalized intensity of all microarray signals.
