# Supplementary material for: Dynamic landscape mapping of humoral immunity to SARS-CoV-2 identifies non-structural protein antibodies associated with the survival of critical COVID-19 patients
Source: Signal Transduct Target Ther. 2021 Aug 17;6:304. doi: 10.1038/s41392-021-00718-w (PMC8368053; doi:10.1038/s41392-021-00718-w)
Supplement: Supplementary file 1 — Supplementary Material [file 41392_2021_718_MOESM1_ESM.docx]

-Supplementary information-

**Dynamic landscape mapping of humoral immunity to SARS-CoV-2 identifies non-structural protein antibodies associated with the survival of critical COVID-19 patients**

Linlin Cheng^1,3#^, Xiaomei Zhang^2,3#^, Yu Chen^1,3#^, Dan Wang^2,3#^, Dong Zhang^1,3#^, Songxin Yan^1,3#^, Hongye Wang^2^, Meng Xiao^1^ ,Te Liang^2^, Haolong Li^1^, Meng Xu^2^, Xin Hou^1^, Jiayu Dai^2^, Xian Wu^1^ , Mingyuan Li^2^, Minya Lu^1^, Dong Wu^1^, Ran Tian^1^, Jing Zhao^1^, Yan Zhang^1^, Wei Cao^1^, Jinglan Wang^1^, Xiaowei Yan^1^, Xiang Zhou^1^, Zhengyin Liu^1^, Yingchun Xu^1^, Fuchu He^2,4^*, Yongzhe Li^1,4^*, Xiaobo Yu^2,4^*, Shuyang Zhang^1,4^*

^1^ Department of Clinical Laboratory, State Key Laboratory of Complex Severe and Rare Diseases, Peking Union Medical College Hospital, Chinese Academy of Medical Science and Peking Union Medical College, Beijing, China.

^2^ State Key Laboratory of Proteomics, Beijing Proteome Research Center, National Center for Protein Sciences-Beijing (PHOENIX Center), Beijing Institute of Lifeomics, Beijing, China.

^3^ These authors contributed equally to this work.

^4^ Correspondence to shuyangzhang103@163.com，yuxiaobo@ncpsb.org.cn, yongzhelipumch@126.com, hefc@nic.bmi.ac.cn

**9 Supplementary Tables**

| Table S1 | Demographics and clinical characteristics of COVID-19 patients in the discovery cohort |
| --- | --- |
| Table S2 | Demographics and clinical characteristics of COVID-19 patients in the validation cohort |
| Table S3 | The layout of the SARS-COV-2 proteome microarray |
| Table S4 | IgM antibody epitopes in critical COVID-19 patients identified with the SARS-COV-2 proteome microarray |
| Table S5 | IgG antibody epitopes in critical COVID-19 patients identified with the SARS-COV-2 proteome microarray |
| Table S6 | References of SARS-CoV-2 protein domains and structures |
| Table S7 | Antibodies associated with COVID-19 prognosis of critical patients |
| Table S8 | Abbreviations and full names of clinical variables |
| Table S9 | Correlations between SARS-CoV-2 proteome antibodies and clinical variables in critical COVID-19 patients |

**26 Supplementary Figures**

| Figure S1 | Serum antibody detection workflow using the SARS-CoV-2 proteome microarray |
| --- | --- |
| Figure S2 | Comparison of antibody responses to SARS-CoV-2 infection in this work and previous studies using proteomics technology. |
| Figure S3 | Comparison of antibody epitopes identified in mild and critical COVID-19 patients. |
| Figure S4 | Spatiotemporal resolution analysis of IgM and IgG antibodies to the SARS-CoV-2 Spike protein. |
| Figure S5 | Structural analysis of the antibody binding epitopes within the S receptor binding domain |
| Figure S6 | Spatiotemporal resolution analysis of antibody binding epitopes within the N protein |
| Figure S7 | Structural analysis of the antibody binding epitopes within the N protein’s RNA binding domain |
| Figure S8 | Distribution of antibody epitopes and glycosylation on the S protein |
| Figure S9 | Longitudinal changes of IgM and IgG antibody binding epitopes within the nsp2 protein |
| Figure S10 | Longitudinal changes and structural analysis of IgM and IgG antibody binding epitopes within the nsp4 protein |
| Figure S11 | Longitudinal changes and structural analysis of IgM and IgG antibody binding epitopes within the nsp6 protein |
| Figure S12 | Spatiotemporal resolution analysis of IgM and IgG antibody binding epitopes within the nsp9 protein |
| Figure S13 | Spatiotemporal resolution analysis of IgM and IgG antibody binding epitopes within the nsp13 protein |
| Figure S14 | Longitudinal changes of IgM and IgG antibody binding epitopes within the nsp14 protein |
| Figure S15 | Spatiotemporal resolution analysis of IgM and IgG antibody binding epitopes within the nsp15 protein. |
| Figure S16 | Longitudinal changes of IgM and IgG antibody binding epitopes within the M protein |
| Figure S17 | Spatiotemporal resolution analysis of IgM and IgG antibody binding epitopes within the ORF3a protein. |
| Figure S18 | Longitudinal changes of IgM and IgG antibody binding epitopes within the ORF7a protein |
| Figure S19 | Spatiotemporal resolution analysis of IgM and IgG antibody binding epitopes within the ORF8 protein |
| Figure S20 | Longitudinal changes of IgM and IgG antibody binding epitopes within the epitopes on the ORF10 protein |
| Figure S21 | Global correlation map of the SARS-CoV-2 proteome IgM antibodies and clinical variables in the non-survival COVID-19 patient group |
| Figure S22 | Global correlation map of the SARS-CoV-2 proteome IgM antibodies and clinical variables in the survival COVID-19 patient group |
| Figure S23 | Global correlation map of the SARS-CoV-2 proteome IgG antibodies and clinical variables in the non-survival COVID-19 patient group |
| Figure S24 | Global correlation map of the SARS-CoV-2 proteome IgG antibodies and clinical variables in the survival COVID-19 patient group |
| Figure S25 | SARS-CoV-2 antibodies in the cluster #1 group |
| Figure S26 | Differential correlations of the clinical variables and SARS-CoV-2 IgM antibodies between survival and non-survival COVID-19 groups in the cluster #2 group |

**Supplementary Tables**

**Tables**

**Table S1. Demographics and clinical characteristics of COVID-19 patients in the discovery cohort.**

|  | Total | Death | Survival | p |
| --- | --- | --- | --- | --- |
| Characteristics |  |  |  |  |
| Number | 49 | 28 | 21 | / |
| Age, years | 66 (57-73) | 65.5 (62-73.75) | 68 (55-71.5) | ns |
| Male | 31 (63.27 %) | 19 (67.86 %) | 12 (57.14 %) | ns |
| Female | 18 (36.73 %) | 9 (32.14 %) | 9 (42.86 %) | ns |
| Smoking history | 6 (12.24 %) | 3 (10.71 %) | 3 (14.29 %) | ns |
| Comorbidities/condition |  |  |  |  |
| Hypertension | 21 (42.86 %) | 11 (39.29 %) | 10 (47.62 %) | ns |
| Diabetes | 9 (18.37 %) | 2 (7.14 %) | 7 (33.33 %) | 0.0488* |
| Cardiovascular disease | 10 (20.41 %) | 5 (17.86 %) | 5 (23.81 %) | ns |
| Cerebrovascular disease | 10 (20.41 %) | 4 (14.29 %) | 6 (28.57 %) | ns |
| Chronic lung disease | 5 (10.20 %) | 3 (10.71 %) | 2 (9.52 %) | ns |
| Chronic liver disease | 3 (6.12 %) | 2 (7.14 %) | 1 (4.76 %) | ns |
| Chronic kidney disease | 4 (8.16 %) | 1 (3.57 %) | 3 (14.29 %) | ns |
| Malignancy | 3 (6.12 %) | 2 (7.14 %) | 1 (4.76 %) | ns |
| Autoimmune diseases | 2 (4.08 %) | 1 (3.57 %) | 1 (4.76 %) | ns |
| Anemia | 1 (2.04 %) | 0 (0.00 %) | 1 (4.76 %) | ns |
| Surgical history | 17 (34.69 %) | 9 (32.14 %) | 8 (38.10 %) | ns |
| Symptoms and signs |  |  |  |  |
| RR ≥ 30 | 35 (71.43 %) | 24 (85.71 %) | 11 (52.38 %) | 0.0106* |
| Fever | 44 (89.80 %) | 26 (92.86 %) | 18 (85.71 %) | ns |
| Fatigue | 22 (44.90 %) | 15 (53.57 %) | 7 (33.33 %) | ns |
| Cough | 35 (71.43 %) | 21 (75.00 %) | 14 (66.67 %) | ns |
| Dyspnea | 31 (63.27 %) | 22 (78.57 %) | 9 (42.86 %) | 0.0103* |
| Headache | 7 (14.29 %) | 5 (17.86 %) | 2 (9.52 %) | ns |
| Abdominal pain | 7 (14.29 %) | 5 (17.86 %) | 2 (9.52 %) | ns |
| Diarrhea | 15 (30.61 %) | 12 (42.86 %) | 3 (14.29 %) | 0.0318* |
| Vomiting | 8 (16.33 %) | 5 (17.86 %) | 3 (14.29 %) | ns |
| Expectoration | 22 (44.90 %) | 12 (42.86 %) | 10 (47.62 %) | ns |
| Treatment |  |  |  |  |
| Glucocorticoid | 31 (63.27 %) | 20 (71.43 %) | 11 (52.38 %) | ns |
| Antibiotic | 47 (95.92 %) | 27 (96.43 %) | 20 (95.24 %) | ns |
| Antiviral | 36 (73.47 %) | 21 (75.00 %) | 15 (71.43 %) | ns |
| Invasive mechanical ventilation | 42 (85.71 %) | 25 (89.29 %) | 17 (80.95 %) | ns |
| CRRT | 11 (22.45 %) | 9 (32.14 %) | 2 (9.52 %) | ns |
| ECMO | 4 (8.16 %) | 4 (14.29 %) | 0 (0.00 %) | ns |
| Outcome |  |  |  |  |
| Shock | 15 (30.61 %) | 12 (42.86 %) | 3 (14.29 %) | 0.0318* |
| ARDS/Respiratory failure | 32 (65.31 %) | 21 (75.00 %) | 11 (52.38 %) | ns |
| Heart failure | 9 (18.37 %) | 6 (21.43 %) | 3 (14.29 %) | ns |
| Acute kidney injury | 8 (16.33 %) | 6 (21.43 %) | 2 (9.52 %) | ns |
| Length of ICU stay, days | 10 (6-18) | 9 (3.25-13) | 11 (8-21) | 0.0471* |
| Time from illness onset to ICU admission, days | 21 (12.5-31) | 18.5 (11.25-25.75) | 25 (15-41.5) | 0.0308* |
| Time from illness onset to first hospital visit, days | 3 (0-7.5) | 3.5 (0-7) | 3 (0-10) | ns |

*Continuous variables were expressed as median (interquartile ranges, IQR) and compared with the t test or Mann-Whitney U test; categorical variables were expressed as a number (%) and compared by a Chi-squared test or Fisher’s exact test between non-survivors and survivors. RR: Respiratory rate; CRRT: Continuous Renal Replacement Therapy; ECMO: Extracorporeal membrane oxygenation; ARDS: Acute respiratory distress syndrome; ICU: Intensive care unit.*

**Table S2. Demographics and clinical characteristics of COVID-19 patients in the validation cohort**

|  | Total | Non-survivor | Survivor | p |
| --- | --- | --- | --- | --- |
| Characteristics |  |  |  |  |
| Number | 22 | 11 | 11 | / |
| Age, years | 65.5 (56.75-71.25) | 68 (57-73) | 65 (55-70) | ns |
| Male | 12 (54.55 %) | 8 (72.73 %) | 4 (36.36 %) | ns |
| Female | 10 (45.45 %) | 3 (27.27 %) | 7 (63.64 %) | ns |
| Smoking history | 1 (4.55 %) | 0 (0.00 %) | 1 (9.09 %) | ns |
| Comorbidities/condition |  |  |  |  |
| Hypertension | 12 (54.55 %) | 6 (54.55 %) | 6 (54.55 %) | ns |
| Diabetes | 4 (18.18 %) | 1 (9.09 %) | 3 (27.27 %) | ns |
| Cardiovascular disease | 6 (27.27 %) | 4 (36.36 %) | 2 (18.18 %) | ns |
| Cerebrovascular disease | 3 (13.64 %) | 1 (9.09 %) | 2 (18.18 %) | ns |
| Chronic lung disease | 1 (4.55 %) | 0 (0.00 %) | 1 (9.09 %) | ns |
| Chronic liver disease | 1 (4.55 %) | 0 (0.00 %) | 1 (9.09 %) | ns |
| Chronic kidney disease | 1 (4.55 %) | 0 (0.00 %) | 1 (9.09 %) | ns |
| Malignancy | 2 (9.09 %) | 0 (0.00 %) | 2 (18.18 %) | ns |
| Autoimmune diseases | 0 (0.00 %) | 0 (0.00 %) | 0 (0.00 %) | ns |
| Anemia | 1 (4.55 %) | 1 (9.09 %) | 0 (0.00 %) | ns |
| Surgical history | 10 (45.45 %) | 4 (36.36 %) | 6 (54.55 %) | ns |
| Symptoms and signs |  |  |  |  |
| RR ≥ 30 | 20 (90.91 %) | 10 (90.91 %) | 10 (90.91 %) | ns |
| Fever | 18 (81.82 %) | 10 (90.91 %) | 8 (72.73 %) | ns |
| Fatigue | 8 (36.36 %) | 4 (36.36 %) | 4 (36.36 %) | ns |
| Cough | 17 (77.27 %) | 10 (90.91 %) | 7 (63.64 %) | ns |
| Dyspnea | 14 (63.64 %) | 7 (63.64 %) | 7 (63.64 %) | ns |
| Headache | 1 (4.55 %) | 1 (9.09 %) | 0 (0.00 %) | ns |
| Abdominal pain | 1 (4.55 %) | 1 (9.09 %) | 0 (0.00 %) | ns |
| Diarrhea | 2 (9.09 %) | 1 (9.09 %) | 1 (9.09 %) | ns |
| Vomiting | 1 (4.55 %) | 1 (9.09 %) | 0 (0.00 %) | ns |
| Expectoration | 9 (40.91 %) | 4 (36.36 %) | 5 (45.45 %) | ns |
| Treatment |  |  |  |  |
| Glucocorticoid | 13 (59.09 %) | 7 (63.64 %) | 6 (54.55 %) | ns |
| Antibiotic | 20 (90.91 %) | 9 (81.82 %) | 11 (100.00 %) | ns |
| Antiviral | 15 (68.18 %) | 9 (81.82 %) | 6 (54.55 %) | ns |
| Invasive mechanical ventilation | 19 (86.36 %) | 10 (90.91 %) | 9 (81.82 %) | ns |
| CRRT | 7 (31.82 %) | 4 (36.36 %) | 3 (27.27 %) | ns |
| ECMO | 2 (9.09 %) | 0 (0.00 %) | 2 (18.18 %) | ns |
| Outcome |  |  |  |  |
| Shock | 11 (50.00 %) | 7 (63.64 %) | 4 (36.36 %) | ns |
| ARDS/Respiratory failure | 17 (77.27 %) | 10 (90.91 %) | 7 (63.64 %) | ns |
| Heart failure | 4 (18.18 %) | 2 (18.18 %) | 2 (18.18 %) | ns |
| Acute kidney injury | 5 (22.73 %) | 3 (27.27 %) | 2 (18.18 %) | ns |
| Length of ICU stay, days | 14 (7.25-21.25) | 10 (2-18) | 16 (8-38) | ns |
| Time from illness onset to ICU admission, days | 23.5 (15.25-34) | 20 (9-22) | 31 (25-62) | 0.0019** |
| Time from illness onset to first hospital visit, days | 4.5 (0-8.25) | 3 (0-8) | 5 (0-9) | ns |

*Continuous variables were expressed as median (interquartile ranges, IQR) and compared with the t test or Mann-Whitney U test; categorical variables were expressed as a number (%) and compared by a Chi-squared test or Fisher’s exact test between non-survivors and survivors. RR: Respiratory rate; CRRT: Continuous Renal Replacement Therapy; ECMO: Extracorporeal membrane oxygenation; ARDS: Acute respiratory distress syndrome; ICU: Intensive care unit.*

**Table S4. IgM antibody epitopes in critical COVID-19 patients identified with the SARS-COV-2 proteome microarray.**

| No. of epitopes | Protein name | Peptide | Epitope ID | Start position | Amino acid sequence | End position |
| --- | --- | --- | --- | --- | --- | --- |
| 1 | nsp2 | ORF1ab-21 | nsp2_26-30 | 26 | ARAGK | 30 |
| 2 | nsp2 | ORF1ab-30 | nsp2_116-120 | 116 | FMGRI | 120 |
| 3 | nsp2 | ORF1ab-72 | nsp2_536-540 | 536 | LYRKC | 540 |
| 4 | nsp3 | ORF1ab-107 | nsp3_248-252 | 248 | KHGGG | 252 |
| 5 | nsp3 | ORF1ab-112 | nsp3_298-302 | 298 | HVVGP | 302 |
| 6 | nsp3 | ORF1ab-141 | nsp3_588-592 | 588 | YKGIK | 592 |
| 7 | nsp3 | ORF1ab-150 | nsp3_678-682 | 678 | TPEEH | 682 |
| 8 | nsp3 | ORF1ab-153 | nsp3_708-712 | 708 | EFLKR | 712 |
| 9 | nsp3 | ORF1ab-155 | nsp3_728-732 | 728 | LDGEV | 732 |
| 10 | nsp3 | ORF1ab-165 | nsp3_828-842 | 828 | YMSALNHTKKWKYPQ | 842 |
|  |  | ORF1ab-166 |  |  |  |  |
| 11 | nsp3 | ORF1ab-175 | nsp3_928-932 | 928 | RVLNV | 932 |
| 12 | nsp3 | ORF1ab-179 | nsp3_968-972 | 968 | PCTCG | 972 |
| 13 | nsp3 | ORF1ab-194 | nsp3_1118-1122 | 1118 | KFADD | 1122 |
| 14 | nsp3 | ORF1ab-197 | nsp3_1148-1202 | 1148 | DVVAIDYKHYTPSFKKGAKLLHKPIVWHVNNATNKATYKPNTWCIRCLWSTKPVE | 1202 |
|  |  | ORF1ab-198 |  |  |  |  |
|  |  | ORF1ab-199 |  |  |  |  |
|  |  | ORF1ab-200 |  |  |  |  |
|  |  | ORF1ab-201 |  |  |  |  |
|  |  | ORF1ab-202 |  |  |  |  |
| 15 | nsp3 | ORF1ab-208 | nsp3_1258-1262 | 1258 | ILKPA | 1262 |
| 16 | nsp3 | ORF1ab-214 | nsp3_1318-1322 | 1318 | DTIAN | 1322 |
| 17 | nsp3 | ORF1ab-219 | nsp3_1368-1372 | 1368 | TNSRI | 1372 |
| 18 | nsp3 | ORF1ab-241 | nsp3_1588-1602 | 1588 | SSTCMMCYKRNRATR | 1602 |
|  |  | ORF1ab-242 |  |  |  |  |
| 19 | nsp3 | ORF1ab-244 | nsp3_1618-1632 | 1618 | VYANGGKGFCKLHNW | 1632 |
|  |  | ORF1ab-245 |  |  |  |  |
| 20 | nsp3 | ORF1ab-252 | nsp3_1698-1702 | 1698 | HSLSH | 1702 |
| 21 | nsp3 | ORF1ab-257 | nsp3_1748-1752 | 1748 | CQPIL | 1752 |
| 22 | nsp3 | ORF1ab-261 | nsp3_1788-1792 | 1788 | MEKLK | 1792 |
| 23 | nsp3 | ORF1ab-266 | nsp3_1838-1842 | 1838 | KLSHQ | 1842 |
| 24 | nsp3 | ORF1ab-269 | nsp3_1868-1872 | 1868 | RDLGA | 1872 |
| 25 | nsp3 | ORF1ab-271 | nsp3_1888-1892 | 1888 | SHNIA | 1892 |
| 26 | nsp3 | ORF1ab-274 | nsp3_1918-1922 | 1918 | NNLPF | 1922 |
| 27 | nsp4 | ORF1ab-277 | nsp4_3-7 | 3 | VNNWL | 7 |
| 28 | nsp4 | ORF1ab-283 | nsp4_63-67 | 63 | CFANK | 67 |
| 29 | nsp4 | ORF1ab-290 | nsp4_133-137 | 133 | CYTPS | 137 |
| 30 | nsp4 | ORF1ab-293 | nsp4_163-167 | 163 | SGKPV | 167 |
| 31 | nsp4 | ORF1ab-301 | nsp4_243-247 | 243 | LNNDY | 247 |
| 32 | nsp4 | ORF1ab-310 | nsp4_333-337 | 333 | PVYSF | 337 |
| 33 | nsp4 | ORF1ab-316 | nsp4_393-397 | 393 | FFSNY | 397 |
| 34 | nsp4 | ORF1ab-321 | nsp4_443-447 | 443 | RYLAL | 447 |
| 35 | nsp5 | ORF1ab-327 | nsp5_3-7 | 3 | FRKMA | 7 |
| 36 | nsp5 | ORF1ab-337 | nsp5_103-107 | 103 | FVRIQ | 107 |
| 37 | nsp5 | ORF1ab-339 | nsp5_123-127 | 123 | SGVYQ | 127 |
| 38 | nsp6 | ORF1ab-358 | nsp6_7-11 | 7 | IKGTH | 11 |
| 39 | nsp6 | ORF1ab-363 | nsp6_57-61 | 57 | MMFVK | 61 |
| 40 | nsp6 | ORF1ab-366 | nsp6_87-91 | 87 | PASWV | 91 |
| 41 | nsp6 | ORF1ab-377 | nsp6_197-201 | 197 | CPIFF | 201 |
| 42 | nsp7 | ORF1ab-394 | nsp7_77-81 | 77 | DNRAT | 81 |
| 43 | nsp8 | ORF1ab-398 | nsp8_34-38 | 34 | VLKKL | 38 |
| 44 | nsp8 | ORF1ab-400 | nsp8_54-58 | 54 | AMQRK | 58 |
| 45 | nsp8 | ORF1ab-402 | nsp8_74-78 | 74 | ARSED | 78 |
| 46 | nsp9 | ORF1ab-420 | nsp9_56-60 | 56 | FPKSD | 60 |
| 47 | nsp9 | ORF1ab-423 | nsp9_86-90 | 86 | KYLYF | 90 |
| 48 | nsp10 | ORF1ab-429 | nsp10_33-37 | 33 | SGGQP | 37 |
| 49 | nsp10 | ORF1ab-434 | nsp10_83-87 | 83 | HPNPK | 87 |
| 50 | nsp10 | ORF1ab-436 | nsp10_103-107 | 103 | CANDP | 107 |
| 51 | nsp10 | ORF1ab-438 | nsp10_123-127 | 123 | WKGYG | 127 |
| 52 | nsp12 | ORF1ab-444 | nsp12_44-48 | 44 | GFAKF | 48 |
| 53 | nsp12 | ORF1ab-447 | nsp12_74-78 | 74 | RHTFS | 78 |
| 54 | nsp12 | ORF1ab-451 | nsp12_114-128 | 114 | ISRQRLTKYTMADLV | 128 |
|  |  | ORF1ab-452 |  |  |  |  |
| 55 | nsp12 | ORF1ab-455 | nsp12_154-158 | 154 | DDYFN | 158 |
| 56 | nsp12 | ORF1ab-461 | nsp12_214-218 | 214 | GNWYD | 218 |
| 57 | nsp12 | ORF1ab-466 | nsp12_264-268 | 264 | PYIKW | 268 |
| 58 | nsp12 | ORF1ab-468 | nsp12_284-288 | 284 | DRYFK | 288 |
| 59 | nsp12 | ORF1ab-472 | nsp12_324-328 | 324 | TSFGP | 328 |
| 60 | nsp12 | ORF1ab-476 | nsp12_364-368 | 364 | SRLSF | 368 |
| 61 | nsp12 | ORF1ab-478 | nsp12_384-388 | 384 | SGNLL | 388 |
| 62 | nsp12 | ORF1ab-485 | nsp12_454-458 | 454 | DYYRY | 458 |
| 63 | nsp12 | ORF1ab-487 | nsp12_474-478 | 474 | EVVDK | 478 |
| 64 | nsp12 | ORF1ab-490 | nsp12_504-508 | 504 | FPFNK | 508 |
| 65 | nsp12 | ORF1ab-493 | nsp12_534-538 | 534 | NVIPT | 538 |
| 66 | nsp12 | ORF1ab-497 | nsp12_574-578 | 574 | KLLKS | 578 |
| 67 | nsp12 | ORF1ab-499 | nsp12_594-608 | 594 | FYGGWHNMLKTVYSD | 608 |
|  |  | ORF1ab-500 |  |  |  |  |
| 68 | nsp12 | ORF1ab-502 | nsp12_624-628 | 624 | RAMPN | 628 |
| 69 | nsp12 | ORF1ab-504 | nsp12_644-658 | 644 | TCCSLSHRFYRLANE | 658 |
|  |  | ORF1ab-505 |  |  |  |  |
| 70 | nsp12 | ORF1ab-510 | nsp12_704-708 | 704 | VNALL | 708 |
| 71 | nsp12 | ORF1ab-512 | nsp12_724-728 | 724 | QHRLY | 728 |
| 72 | nsp12 | ORF1ab-514 | nsp12_744-748 | 744 | EFYAY | 748 |
| 73 | nsp12 | ORF1ab-520 | nsp12_804-808 | 804 | DLTKG | 808 |
| 74 | nsp12 | ORF1ab-531 | nsp12_914-918 | 914 | RYWEP | 918 |
| 75 | nsp13 | ORF1ab-535 | nsp13_22-36 | 22 | RPFLCCKCCYDHVIS | 36 |
|  |  | ORF1ab-536 |  |  |  |  |
| 76 | nsp13 | ORF1ab-540 | nsp13_72-76 | 72 | CKSHK | 76 |
| 77 | nsp13 | ORF1ab-549 | nsp13_162-166 | 162 | ELHLS | 166 |
| 78 | nsp13 | ORF1ab-557 | nsp13_242-246 | 242 | PQEHY | 246 |
| 79 | nsp13 | ORF1ab-560 | nsp13_272-276 | 272 | VGMQK | 276 |
| 80 | nsp13 | ORF1ab-565 | nsp13_322-326 | 322 | LKYLP | 326 |
| 81 | nsp13 | ORF1ab-567 | nsp13_342-346 | 342 | CFDKF | 346 |
| 82 | nsp13 | ORF1ab-572 | nsp13_392-396 | 392 | RAKHY | 396 |
| 83 | nsp13 | ORF1ab-575 | nsp13_422-426 | 422 | FNSVC | 426 |
| 84 | nsp13 | ORF1ab-577 | nsp13_442-446 | 442 | RRCPA | 446 |
| 85 | nsp13 | ORF1ab-579 | nsp13_462-476 | 462 | KAHKDKSAQCFKMFY | 476 |
|  |  | ORF1ab-580 |  |  |  |  |
| 86 | nsp13 | ORF1ab-583 | nsp13_502-506 | 502 | RNPAW | 506 |
| 87 | nsp13 | ORF1ab-591 | nsp13_582-586 | 582 | YDKLQ | 586 |
| 88 | nsp14 | ORF1ab-594 | nsp14_11-15 | 11 | CSKVI | 15 |
| 89 | nsp14 | ORF1ab-598 | nsp14_51-55 | 51 | YRRLI | 55 |
| 90 | nsp14 | ORF1ab-608 | nsp14_151-155 | 151 | PLMYK | 155 |
| 91 | nsp14 | ORF1ab-624 | nsp14_311-325 | 311 | KVQHMVVKAALLADK | 325 |
|  |  | ORF1ab-625 |  |  |  |  |
| 92 | nsp14 | ORF1ab-627 | nsp14_341-345 | 341 | VPQAD | 345 |
| 93 | nsp14 | ORF1ab-632 | nsp14_391-395 | 391 | RYPAN | 395 |
| 94 | nsp14 | ORF1ab-635 | nsp14_421-435 | 421 | VNKHAFHTPAFDKSA | 435 |
|  |  | ORF1ab-636 |  |  |  |  |
| 95 | nsp14 | ORF1ab-641 | nsp14_481-485 | 481 | GAVCR | 485 |
| 96 | nsp15 | ORF1ab-650 | nsp15_44-68 | 44 | ENKTTLPVNVAFELWAKRNIKPVPE | 68 |
|  |  | ORF1ab-651 |  |  |  |  |
|  |  | ORF1ab-652 |  |  |  |  |
| 97 | nsp15 | ORF1ab-671 | nsp15_254-258 | 254 | LAKRF | 258 |
| 98 | nsp16 | ORF1ab-698 | nsp16_178-182 | 178 | ADLYK | 182 |
| 99 | nsp16 | ORF1ab-705 | nsp16_248-252 | 248 | SKFPL | 252 |
| 100 | nsp16 | ORF1ab-708 | nsp16_278-282 | 278 | GRLII | 282 |
| 101 | S | S-4 | S_36-40 | 36 | VYYPD | 40 |
| 102 | S | S-36 | S_356-360 | 356 | KRISN | 360 |
| 103 | S | S-43 | S_426-430 | 426 | PDDFT | 430 |
| 104 | S | S-56 | S_556-560 | 556 | NKKFL | 560 |
| 105 | S | S-82 | S_816-820 | 816 | SFIED | 820 |
| 106 | S | S-98 | S_976-980 | 976 | VLNDI | 980 |
| 107 | S | S-109 | S_1086-1090 | 1086 | KAHFP | 1090 |
| 108 | ORF3a | ORF3a-7 | ORF3a_66-70 | 66 | KKRWQ | 70 |
| 109 | ORF3a | ORF3a-14 | ORF3a_136-140 | 136 | KNPLL | 140 |
| 110 | M | M-1 | M_6-10 | 6 | GTITV | 10 |
| 111 | M | M-15 | M_146-150 | 146 | RGHLR | 150 |
| 112 | ORF7a | ORF7a-12 | ORF7a_116-121 | 116 | LKRKTE | 121 |
| 113 | ORF8 | ORF8-5 | ORF8_46-50 | 46 | YIRVG | 50 |
| 114 | N | N-10 | N_96-100 | 96 | GGDGK | 100 |
| 115 | N | N-17 | N_166-170 | 166 | TLPKG | 170 |
| 116 | N | N-21 | N_206-210 | 206 | SPARM | 210 |
| 117 | N | N-25 | N_246-250 | 246 | VTKKS | 250 |
| 118 | N | N-37 | N_366-370 | 366 | TEPKK | 370 |

**Table S5. IgG antibody epitopes in critical COVID-19 patients identified with the SARS-COV-2 proteome microarray.**

| No. of epitopes | Protein name | Peptide ID | Epitope ID | Start position | Amino acid sequence | End position |
| --- | --- | --- | --- | --- | --- | --- |
| 1 | nsp1 | ORF1ab-12 | nsp1_116-130 | 116 | VAYRKVLLRKNGNKG | 130 |
|  |  | ORF1ab-13 |  |  |  |  |
| 2 | nsp1 | ORF1ab-16 | nsp1_156-170 | 156 | DFQENWNTKHSSGVT | 170 |
|  |  | ORF1ab-17 |  |  |  |  |
| 3 | nsp2 | ORF1ab-29 | nsp2_106-110 | 106 | PRVEK | 110 |
| 4 | nsp2 | ORF1ab-40 | nsp2_216-220 | 216 | ILRKG | 220 |
| 5 | nsp2 | ORF1ab-63 | nsp2_446-450 | 446 | PVLDW | 450 |
| 6 | nsp3 | ORF1ab-112 | nsp3_298-302 | 298 | HVVGP | 302 |
| 7 | nsp3 | ORF1ab-135 | nsp3_528-532 | 528 | LKKCK | 532 |
| 8 | nsp3 | ORF1ab-140 | nsp3_578-582 | 578 | KAIVS | 582 |
| 9 | nsp3 | ORF1ab-153 | nsp3_708-712 | 708 | EFLKR | 712 |
| 10 | nsp3 | ORF1ab-165 | nsp3_828-842 | 828 | YMSALNHTKKWKYPQ | 842 |
|  |  | ORF1ab-166 |  |  |  |  |
| 11 | nsp3 | ORF1ab-197 | nsp3_1148-1152 | 1148 | DVVAI | 1152 |
| 12 | nsp3 | ORF1ab-202 | nsp3_1198-1202 | 1198 | TKPVE | 1202 |
| 13 | nsp3 | ORF1ab-219 | nsp3_1368-1372 | 1368 | TNSRI | 1372 |
| 14 | nsp3 | ORF1ab-251 | nsp3_1688-1692 | 1688 | DKAGQ | 1692 |
| 15 | nsp3 | ORF1ab-266 | nsp3_1838-1842 | 1838 | KLSHQ | 1842 |
| 16 | nsp4 | ORF1ab-277 | nsp4_3-7 | 3 | VNNWL | 7 |
| 17 | nsp4 | ORF1ab-321 | nsp4_443-447 | 443 | RYLAL | 447 |
| 18 | nsp5 | ORF1ab-336 | nsp5_93-97 | 93 | TANPK | 97 |
| 19 | nsp6 | ORF1ab-358 | nsp6_7-11 | 7 | IKGTH | 11 |
| 20 | nsp6 | ORF1ab-384 | nsp6_267-271 | 267 | DAFKL | 271 |
| 21 | nsp9 | ORF1ab-420 | nsp9_56-60 | 56 | FPKSD | 60 |
| 22 | nsp9 | ORF1ab-423 | nsp9_86-90 | 86 | KYLYF | 90 |
| 23 | nsp10 | ORF1ab-435 | nsp10_93-97 | 93 | KGKYV | 97 |
| 24 | nsp12 | ORF1ab-447 | nsp12_74-78 | 74 | RHTFS | 78 |
| 25 | nsp12 | ORF1ab-452 | nsp12_124-128 | 124 | MADLV | 128 |
| 26 | nsp12 | ORF1ab-466 | nsp12_264-268 | 264 | PYIKW | 268 |
| 27 | nsp12 | ORF1ab-485 | nsp12_454-458 | 454 | DYYRY | 458 |
| 28 | nsp12 | ORF1ab-487 | nsp12_474-478 | 474 | EVVDK | 478 |
| 29 | nsp12 | ORF1ab-490 | nsp12_504-508 | 504 | FPFNK | 508 |
| 30 | nsp12 | ORF1ab-495 | nsp12_554-558 | 554 | ARTVA | 558 |
| 31 | nsp12 | ORF1ab-499 | nsp12_594-598 | 594 | FYGGW | 598 |
| 32 | nsp12 | ORF1ab-514 | nsp12_744-748 | 744 | EFYAY | 748 |
| 33 | nsp13 | ORF1ab-535 | nsp13_22-26 | 22 | RPFLC | 26 |
| 34 | nsp13 | ORF1ab-550 | nsp13_172-176 | 172 | PRPPL | 176 |
| 35 | nsp13 | ORF1ab-565 | nsp13_322-326 | 322 | LKYLP | 326 |
| 36 | nsp13 | ORF1ab-579 | nsp13_462-466 | 462 | KAHKD | 466 |
| 37 | nsp13 | ORF1ab-583 | nsp13_502-506 | 502 | RNPAW | 506 |
| 38 | nsp14 | ORF1ab-636 | nsp14_431-435 | 431 | FDKSA | 435 |
| 39 | nsp15 | ORF1ab-652 | nsp15_64-68 | 64 | KPVPE | 68 |
| 40 | nsp15 | ORF1ab-671 | nsp15_254-258 | 254 | LAKRF | 258 |
| 41 | S | S-3 | S_26-40 | 26 | PAYTNSFTRGVYYPD | 40 |
|  |  | S-4 |  |  |  |  |
| 42 | S | S-15 | S_146-150 | 146 | HKNNK | 150 |
| 43 | S | S-19 | S_186-190 | 186 | FKNLR | 190 |
| 44 | S | S-36 | S_356-360 | 356 | KRISN | 360 |
| 45 | S | S-45 | S_446-460 | 446 | GGNYNYLYRLFRKSN | 460 |
|  |  | S-46 |  |  |  |  |
| 46 | S | S-56 | S_556-560 | 556 | NKKFL | 560 |
| 47 | S | S-58 | S_576-580 | 576 | VRDPQ | 580 |
| 48 | S | S-68 | S_676-680 | 676 | TQTNS | 680 |
| 49 | S | S-80 | S_796-830 | 796 | DFGGFNFSQILPDPSKPSKRSFIEDLLFNKVTLAD | 830 |
|  |  | S-81 |  |  |  |  |
|  |  | S-82 |  |  |  |  |
|  |  | S-83 |  |  |  |  |
| 50 | S | S-126 | S_1256-1268 | 1256 | FDEDDSEPVLKGV | 1268 |
|  |  | S-127 |  |  |  |  |
| 51 | ORF3a | ORF3a-14 | ORF3a_136-140 | 136 | KNPLL | 140 |
| 52 | ORF3a | ORF3a-18 | ORF3a_176-190 | 176 | TSPISEHDYQIGGYT | 190 |
|  |  | ORF3a-19 |  |  |  |  |
| 53 | ORF3a | ORF3a-22 | ORF3a_216-220 | 216 | STQLS | 220 |
| 54 | ORF3a | ORF3a-26 | ORF3a_256-260 | 256 | VNPVM | 260 |
| 55 | M | M-11 | M_106-110 | 106 | TRSMW | 110 |
| 56 | M | M-17 | M_166-170 | 166 | KEITV | 170 |
| 57 | ORF7a | ORF7a-12 | ORF7a_116-121 | 116 | LKRKTE | 121 |
| 58 | ORF8 | ORF8-1 | ORF8_6-10 | 6 | FLGII | 10 |
| 59 | ORF8 | ORF8-4 | ORF8_36-50 | 36 | PCPIHFYSKWYIRVG | 50 |
|  |  | ORF8-5 |  |  |  |  |
| 60 | N | N-4 | N_36-40 | 36 | RSKQR | 40 |
| 61 | N | N-7 | N_66-70 | 66 | FPRGQ | 70 |
| 62 | N | N-10 | N_96-110 | 96 | GGDGKMKDLSPRWYF | 110 |
|  |  | N-11 |  |  |  |  |
| 63 | N | N-13 | N_126-130 | 126 | NKDGI | 130 |
| 64 | N | N-17 | N_166-170 | 166 | TLPKG | 170 |
| 65 | N | N-23 | N_226-250 | 226 | RLNQLESKMSGKGQQQQGQTVTKKS | 250 |
|  |  | N-24 |  |  |  |  |
|  |  | N-25 |  |  |  |  |
| 66 | N | N-27 | N_266-270 | 266 | KAYNV | 270 |
| 67 | N | N-32 | N_316-320 | 316 | GMSRI | 320 |
| 68 | N | N-35 | N_346-400 | 346 | FKDQVILLNKHIDAYKTFPPTEPKKDKKKKADETQALPQRQKKQQTVTLLPAADL | 400 |
|  |  | N-36 |  |  |  |  |
|  |  | N-37 |  |  |  |  |
|  |  | N-38 |  |  |  |  |
|  |  | N-39 |  |  |  |  |
|  |  | N-40 |  |  |  |  |
| 69 | ORF10 | ORF10-3 | ORF10_26-33 | 26 | YIAQVDVV | 33 |

**Table S6. References of SARS-CoV-2 protein domains and structures.**

| **Protein** | **PDB** | **PMID** | **Domain** | **PMID** |
| --- | --- | --- | --- | --- |
| **nsp1** | 6ZOK | 32908316 | N-terminal domain (a.a.1-128) | 32680882;32908316; 33037187;32995777 |
|  |  |  | C-terminal domain (a.a.148-180) | 32680882;32908316; 33037187;32995777 |
| **nsp2** | None | None | Predicted transmembrane helices 1 (a.a.223-228) | 32083328 |
|  |  |  | Predicted transmembrane helices 2 (a.a.349-364) | 32083328 |
|  |  |  | Predicted transmembrane helices 3 (a.a.400-430) | 32083328 |
|  |  |  | Predicted transmembrane helices 4 (a.a.452-482) | 32083328 |
| **nsp3** | 6W6Y | 32939273 | Macrodomain domain (Mac1, also known as ADP-ribose phosphatase domain, ADRP; a.a.207-377) | 32578982;32981460 |
|  | 6W9C | None | Papain-Like cysteine protease domain (PLpro, a.a 746-1063) | 32845033;32895623; 32726803 |
| **nsp4** | None | None | Putative transmembrane domain 1 (TM1, a.a.13-35) | 28738245 |
|  |  |  | Putative transmembrane domain 2 (TM2, a.a.280-302) | 28738245 |
|  |  |  | Putative transmembrane domain 3 (TM3, a.a.315-337) | 28738245 |
|  |  |  | Putative transmembrane domain 4 (TM4, a.a.365-387) | 28738245 |
| **nsp5** | 6Y2G | 32198291 | N-Finger (a.a.1-7) | 32382072;32198291; 33028810;32296570 |
|  |  |  | Domain I (a.a.8-101) | 32382072;32198291; 33028810;32296570 |
|  |  |  | Domain II (a.a.102-184) | 32382072;32198291; 33028810;32296570 |
|  |  |  | Domain III (a.a.201-303) | 32382072;32198291; 33028810;32296570 |
|  |  |  | Loop (a.a.45-53) | 32382072;32198291; 33028810;32296570 |
|  |  |  | Loop (a.a.185-200) | 32382072;32198291; 33028810;32296570 |
|  |  |  | His41, Cys145 | 32382072;32198291; 33028810;32296570 |
| **nsp6** | None | None | Putative transmembrane domain 1 (TM1, a.a.19-38) | 32965508 |
|  |  |  | Putative transmembrane domain 2 (TM2, a.a.42-60) | 32965508 |
|  |  |  | Putative transmembrane domain 3 (TM3, a.a.64-94) | 32965508 |
|  |  |  | Putative transmembrane domain 4 (TM4, a.a.104-131) | 32965508 |
|  |  |  | Putative transmembrane domain 5 (TM5, a.a.136-154) | 32965508 |
|  |  |  | Putative transmembrane domain 6 (TM6, a.a.162-179) | 32965508 |
|  |  |  | Putative transmembrane domain 7 (TM7, a.a.183-203) | 32965508 |
|  |  |  | Putative transmembrane domain 8 (TM8, a.a.207-237) | 32965508 |
| **nsp7** | 6YYT | 32438371 | None | None |
| **nsp8** | 6YYT | 32438371 | Extension (a.a.1-98) | 32783916 |
|  |  |  | Head (a.a.99-198) | 32783916 |
| **nsp9** | 6WXD | 32592996 | None | None |
| **nsp10** | 7C2J | 32728018 | None | None |
| **nsp12** | 6YYT | 32438371 | Nnidovirus RdRp-associated nucleotidyltransferase domain (NiRA; a.a.1-250) | 32783916;32438371 |
|  |  |  | Interface domain (a.a. 251-398) | 32783916;32438371 |
|  |  |  | RNA-dependent RNA polymerase domain (RdRp; comprise the fingers (a.a.399-581,528-687), palm (a.a.582-627,688-812) and thumb (a.a.813-912) sub domains | 32783916;32438371 |
| **nsp13** | 6XEZ | 32783916 | N-terminal Zinc binding domain (ZBD) (a.a.1–100) | 32817950;32783916 |
|  |  |  | stalk domain (a.a.101–149, 230-234) | 32817950;32783916 |
|  |  |  | 1B domain (a.a.150–229) | 32817950;32783916 |
|  |  |  | “RecA-like” domains 1 (a.a. 234–439) | 32817950;32783916 |
|  |  |  | “RecA-like” domains 2 (a.a. 440–601) | 32817950;32783916 |
| **nsp14** | None | None | C-terminal N7-Mtase domain (a.a.301-527) | 32923004 |
|  |  |  | N-terminal 3′-5′ ExoN domain (a.a. 1-287) | 32923004 |
| **nsp15** | 6VWW | 32304108 | N-terminal domain (ND, a.a.1-62) | 32304108;32803198 |
|  |  |  | Middle domain (MD, a.a.63-191) | 32304108;32803198 |
|  |  |  | C terminal catalytic NendoU domain (endoU, a.a.192-346) | 32304108;32803198 |
| **nsp16** | 7C2J | 32728018 | None | None |
| **S** | 6VXX | 32155444 | N-terminal domain (NTD, a.a. 14-306) | 32694201;32320687; 32275855;32225176; 32155444 |
|  |  |  | Receptor-binding domain (RBD, a.a. 331–527) | 32694201;32320687; 32275855;32225176; 32155444 |
|  |  |  | C-terminal domain 1 (CTD1) | 32694201;32320687; 32275855;32225176; 32155444 |
|  |  |  | C-terminal domain 2 (CTD2, a.a. 307–685) | 32694201;32320687; 32275855;32225176; 32155444 |
|  |  |  | Connector domain  (CD, a.a.1035-1067) | 32694201;32320687; 32275855;32225176; 32155444 |
|  |  |  | Transmembrane anchor (TM, a.a. 1211-1233) | 32694201;32320687; 32275855;32225176; 32155444 |
|  |  |  | Fusion peptide and the fusion peptide proximal region (FP and FPPR, a.a.816-833) | 32694201;32320687; 32275855;32225176; 32155444 |
|  |  |  | Heptad repeat 1 (HR1, a.a.910-984) | 32694201;32320687; 32275855;32225176; 32155444 |
|  |  |  | Heptad repeat 2 (HR2, a.a.1163-1210) | 32694201;32320687; 32275855;32225176; 32155444 |
|  |  |  | Central helix region (CH, a.a.985-1034) | 32694201;32320687; 32275855;32225176; 32155444 |
|  |  |  | Cytoplasmic tail (CT, a.a.1234-1273) | 32694201;32320687; 32275855;32225176; 32155444 |
|  |  |  | S1/S2 furin cleavage site (S1/S2, R685↓S686) | 32694201;32320687; 32275855;32225176; 32155444 |
| **ORF3a** | 6XDC | None | Domain I: N terminus putative signal peptide (aa 1-15) | 32371472;32587976 |
|  |  |  | Domain II: TRAF3-binding motif (aa 36-40) | 32371472;32587976 |
|  |  |  | Domain III: A K ion channel (aa 91-133) | 32371472;32587976 |
|  |  |  | Domain IV: a caveolin-binding motif (aa 141-149) | 32371472;32587976 |
|  |  |  | Domain V: The YXXΦ motif (aa 160-163) | 32371472;32587976 |
|  |  |  | Domain VI: a diacidic motif (aa 171-173) | 32371472;32587976 |
|  |  |  | Transmembrane region 1(TM1: a.a. 42-62) | 32371472;32587976 |
|  |  |  | Transmembrane region 2(TM1: a.a. 70-98) | 32371472;32587976 |
|  |  |  | Transmembrane region 3(TM1: a.a. 104-132) | 32371472;32587976 |
| **E** | None | None | None | None |
| **M** | None | None | Transmembrane helices 1  (TM1, a.a. 22-37) | 32596311 |
|  |  |  | Transmembrane helices 2  (TM2, a.a. 48-67) | 32596311 |
|  |  |  | Transmembrane helices 3  (TM3, a.a. 77-97) | 32596311 |
| **ORF6** | None | None | None | None |
| **ORF7a** | 6W37 | None | N-terminal  signal peptide (a.a.1-15) | 15642263;26378163 |
|  |  |  | Luminal domain (a.a.16-95) | 15642263;26378163 |
|  |  |  | Transmembrane  segment (a.a. 96-116) | 15642263;26378163 |
|  |  |  | Cytoplasmic tail (a.a.117-121) | 15642263;26378163 |
| **ORF8** | 7JTL | 32869027 | Signal peptide (a.a.1-16) | 32825438 |
| **N** | NTD:  7CDZ | None | N-terminal domain / RNA-binding domain (NTD, a.a. 44-174) | 33095454;32914439; 32416961 |
|  | CTD:  7CE0 | None | C terminal domain (CTD, a.a.255-364) | 33095454;32914439; 32416961 |
|  | RBD:  6M3M | 32363136 | Linker region (LKR, a.a. 175-254) | 33095454;32914439; 32416961 |
| **ORF10** | None | None | α-helical (a.a.3-21) | 32353859 |

**Table S8. Abbreviation and full names of clinical variables.**

| **No.** | **Abbreviation** | **Full name** |
| --- | --- | --- |
| 1 | Alb | Albumin |
| 2 | ALP | Alkaline phosphatase |
| 3 | ALT | Alanine aminotransferase |
| 4 | APTT | Active partial thromboplastin time |
| 5 | AST | Aspartate aminotransferase |
| 6 | BASO | Basophil |
| 7 | BASOp | The percentage of basophils |
| 8 | Ca | Calcium |
| 9 | Ca_adj | Adjusted calcium |
| 10 | CKMB | Creatine kinase-MB |
| 11 | Cl | Chlorine |
| 12 | CR | Creatinine |
| 13 | DBIL | Direct bilirubin |
| 14 | eGFR | Estimated glomerular filtration rate |
| 15 | EOS | Eosinophil |
| 16 | EOSp | The percentage of eosinophils |
| 17 | FG | Fibrinogen |
| 18 | GGT | Gamma glutamyl transferase |
| 19 | Glb | Globulin |
| 20 | Glu | Glucose |
| 21 | HCO3 | HCO3- |
| 22 | HCT | Hematocrit |
| 23 | HGB | Hemoglobin |
| 24 | hs_CRP | High-sensitivity C-reactive protein |
| 25 | hs_cTnI | High-sensitivity troponin I |
| 26 | IBIL | Indirect bilirubin |
| 27 | INR | International normalized ratio |
| 28 | K | Potassium |
| 29 | LDH | Lactate dehydrogenase |
| 30 | LY | Lymphocyte |
| 31 | Lyp | The percentage of lymphocytes |
| 32 | Mb | Myoglobin |
| 33 | MCH | Mean corpuscular hemoglobin |
| 34 | MCHC | Mean corpuscular hemoglobin concentration |
| 35 | MCV | Mean corpuscular volume |
| 36 | MONO | Monocyte |
| 37 | MONOp | The percentage of monocytes |
| 38 | MPV | Mean platelet volume |
| 39 | Na | Sodium |
| 40 | NEUT | Neutrophil |
| 41 | NEUTp | The percentage of neutrophils |
| 42 | NT_proBNP | N-terminal pro b-type natriuretic peptide |
| 43 | P_LCR | Platelet-large cell ratio |
| 44 | pCO2 | Partial pressure of carbon dioxide |
| 45 | PDW | Platelet distribution width |
| 46 | PLT | Platelet |
| 47 | pO2 | Partial pressure of oxygen |
| 48 | PT | Prothrombin time |
| 49 | PTA | Prothrombin activity |
| 50 | RBC | Red blood cell |
| 51 | RDW_CV | Red cell volume distribution width (coefficient of variation) |
| 52 | RDW_SD | Red cell volume distribution width (standard deviation) |
| 53 | TBIL | Total bilirubin |
| 54 | TC | Total cholesterol |
| 55 | TP | Total protein |
| 56 | TT | Thrombin time |
| 57 | UA | Uric acid |
| 59 | WBC | White blood cell |

**Supplementary Text**

**Distribution of antibody epitopes on SARS-CoV-2 structural proteins**

**1. Spike (S) protein**

The immunogenicity of the S protein was previously well characterized (Supplementary Fig. 2a, Supplementary Table 6). Three epitopes within the receptor binding domain (S_456-460, S_556-560, S_816-820) were reported as the targets of neutralizing antibodies (Supplementary Fig. 4b, 4c and 5, Supplementary Tables 4-5) ^1-3^. During viral entry, the S protein trimerizes. As such, structural analyses of the trimerized S protein were performed to identify the locations of the epitopes within the S trimerized structure. The analyses indicate that some epitopes of IgM antibodies (S_356-360, S_556-560, S_816-820) and IgG antibodies (S_26-40, S_186-190, S_356-360, S_446-460, S_556-560, S_676-680, S_796-830) are located on the surface of the S trimer, while other epitopes (IgM antibodies: S_36-40, S_426-430, S_976-980, S_1086-1090) (IgG antibodies: S_576-580) are located inside the structure (Supplementary Fig. 2b and 2c).

1. **Nucleocapsid (N) protein**

Antibody epitopes are distributed across the entire N protein sequence (Fig. 3c). However, unlike the S protein, high levels of IgM and IgG antibodies targeted only two N protein epitopes (N_206-210 and N_166-170, respectively) (Fig. 3c, red arrow). Notably, IgG antibodies to the N_226-250 epitope were highly expressed in patients who did not survive (Fig. 3c, blue arrow), suggesting that there may be an association between SARS-CoV-2 antibodies and COVID-19 mortality. Structural analyses further indicate that all IgM and IgG epitopes are distributed on the surface of the N protein (Supplementary Fig. 6-7). Notably, two epitopes (N_96-100, N_166-170) located within the N-terminal domain (NTD) (Fig. 3d) were targeted by both IgM and IgG antibodies. However, six epitopes were targeted only by IgG antibodies. These epitopes included three epitopes (N_66-70, N_101-110, N_126-130) on the NTD and three epitopes (N_266-270, N_316-320, N_346-364) on the C-terminal domain (CTD) (Fig. 3e, Supplementary Tables 4-5).

1. **Envelop (E) protein**

The E protein is a small integral membrane protein in coronaviruses, which can oligomerize and create an ion channel ^4^. No antibody epitopes were identified in this work as well as our previous study analyzing antibody epitopes of early COVID-19 patients ^3^. These results are in accordance with previous studies using VirScan and protein microarrays ^5^.

1. **Membrane (M) protein**

The M protein is an integral membrane protein with three transmembrane (TM) domains and is important in viral assembly through its interaction with other viral proteins ^4^. Our epitope mapping study identified two IgM epitopes (M_6-10 and M_146-150) and two IgG epitopes (M_106-110 and M_166-170) in critical COVID-19 patients (Supplementary Tables 4 and 5). The structural domain analysis further indicated that one epitope (M_6-10) is located at the N-terminus while the other three epitopes are located at the C-terminus; none of the epitopes are located within the TM domains (Supplementary Fig. 16).

**Distribution of antibody epitopes on SARS-CoV-2 accessory proteins**

1. **ORF3a protein**

The ORF3a protein is a multi-channel membrane protein (275 amino acids) containing six functional domains, including a signaling peptide, TRAF3 binding motif, ion channels, caveolin-binding motif, YxxΦ motif, and a Di-acidic motif. Our epitope mapping identified two IgM epitopes (ORF3a_66-70, ORF3a_136-140) and four IgG epitopes (ORF3a_136-140, ORF3a_176-190, ORF3a_216-220, ORF3a_256-260) (Supplementary Fig. 17a, Supplementary Tables 4 and 5). All these epitopes are located outside of ion channel in the cytosol (Supplementary Fig. 17b and 17c) Interestingly, ORF3a is released from SARS infected cells. It is possible that the humoral immunity to ORF3a neutralizes viral activity by recognizing SARS-CoV-2 accessory proteins. Indeed, ORF3a from SARS-CoV-2 is associated with cell apoptosis ^6^.

1. **ORF6 protein**

The ORF6 protein exhibits inhibition activity to the type I interferon signaling pathway ^7,8^. No antibody epitope to the ORF6 was identified in this study.

1. **ORF7a protein**

The ORF7a protein is a short type I transmembrane protein (121 amino acids) and modulates BST2/tetherin for immune invasion ^9^. In this work, only one epitope (ORF7a_116-121) was identified, which is located within the cytoplasmic tail (Supplementary Fig. 18). Both IgM and IgG antibodies targeted this epitope.

1. **ORF8 protein**

The ORF8 protein is a rapidly evolving accessory protein that has been proposed to interfere with immune responses ^10^. ORF8 possesses a signaling peptide for importing into the host’s endoplasmic reticulum. In this work, an IgM epitope (ORF8_46-50) and two IgG epitopes (ORF8_6-10, ORF8_36-50) were identified (Supplementary Fig. 19a, Supplementary Tables 4 and 5). The structural analysis indicated that the ORF8_6-10 epitope is located within the signaling peptide while the ORF8_46-50 epitope is located on the loop of protein (Supplementary Fig. 19b and 19c).

1. **ORF10 protein**

ORF10 is a small protein (38 amino acids) with an unknown function. Our data identified an IgG epitope (ORF10_26-33) that is located at the C-terminus (Supplementary Fig. 20).

**Distribution of antibody epitopes on SARS-CoV-2 non-structural proteins**

1. **Nsp2 protein**

The function of nsp2 in COVID-19 pathogenesis is unknown. However, the nsp2 protein is predicted to have four transmembrane helices ^11^. In this work, three IgM epitopes (nsp2_26-30, nsp2_116-120, nsp2_536-540) and three IgG epitopes (nsp2_106-110, nsp2_216-220, nsp2_446-450) were identified (Supplementary Fig. 9, Supplementary Tables 4 and 5). From the predicted structural domain, two IgM epitopes (nsp2_26-30, nsp2_116-120) and two IgG epitopes (nsp2_106-110, nsp2_216-220) are located at the front of transmembrane helices in the extracellular environment. The IgM epitope (nsp2_536-540) is located after the transmembrane helices in the extracellular environment. The IgG epitope (nsp2_446-450) is located between the third and fourth helices in the cytosol (Supplementary Fig. 9).

1. **Nsp4 protein**

The sequence identity of SARS-CoV-2 nsp4 is 80% homologous to SARS-CoV’s nsp4 protein. In SARS-CoV, nsp4 has a multi transmembrane domain and is involved in the membrane rearrangement and viral replication by interaction with nsp3^4^. In this work, 8 IgM epitopes and 2 IgG epitopes were identified within the nsp4 protein (Supplementary Tables 4 and 5). The IgM and IgG epitope (nsp4_3-7) is located at the front of transmembrane domain in the cytoplasmic side. Four IgM epitopes (nsp4_63-67, nsp4_133-137, nsp4_163-167, nsp4_243-247) are located within the luminal side between the first and second transmembrane domains. Two epitopes (nsp4_393-397 and nsp4_443-447) are located within C-terminal intracellular domain (Supplementary Fig. 10).

1. **Nsp6 protein**

Nsp6, a membrane protein, is involved in membrane rearrangement for viral replication and interacts with sigma receptors to block ER-induced autophagosome/autolysosome vesicle that restricts viral production ^12^. Haloperidol, which binds strongly to nsp6, has been reported as a candidate drug against COVID-19^12^. In this work, four IgM epitopes (nsp6_7-11, nsp6_57-61, nsp6_87-91, nsp6_197-201) and two IgG epitopes (nsp6_7-11, nsp6_267-271) were identified by epitope mapping (Supplementary Fig. 11). From the predicted nsp6 protein structure, the majority of IgM epitopes (nsp6_57-61, nsp6_87-91, nsp6_197-201) are within the transmembrane domains, which was not observed with other SARS-CoV-2 proteins in this study. Two IgG epitopes (nsp6_7-11, nsp6_267-271) are located outside of the transmembrane domains (Supplementary Fig. 11).

1. **Nsp9 protein**

SARS nsp9 forms dimer via a conserved a-helical ‘‘100-GxxxG-105’’ motif in solution, and the interruption of key residues within this motif may suppress RNA binding and viral replication ^13^. In this work, two epitopes (nsp9_56-60, nsp9_86-90) were identified for both IgM and IgG antibodies (Supplementary Fig. 12a, Supplementary Tables 4 and 5). The epitopes are distributed on the surface of the Nsp9 dimer (Supplementary Fig. 12b).

1. **Nsp13 protein**

Nsp13 is a helicase and can generate a SARS-CoV-2 helicase-replication transcription complex with the accessory proteins, nsp7, nsp8, and nsp12, which is essential during the transcription and replication of viral genome. In this work, 13 IgM and 5 IgG epitopes were identified, which are distributed across the entire protein sequence and on the surface of protein (Supplementary Fig. 13). However, antibody epitopes on the interface of the nsp13/RdRp complex were not observed.

1. **Nsp14 protein**

The bifunctional ssp14 has 3′-to-5′ exoribonuclease (ExoN) and guanine-N7-methyltransferase (N7-MTase) domains, which are used to mediate proofreading during genome replication and mRNA capping, respectively^14^. In this work, 8 IgM epitopes (nsp14_11-15, nsp14_51-55, nsp14_151-155, nsp14_311-325, nsp14_341-345, nsp14_391-395, nsp14_421-435, nsp14_481-485) and 1 IgG epitope (nsp14_431-435) were identified (Supplementary Tables 4 and 5). The structural analysis indicate that two IgM epitopes (nsp14_311-325, nsp14_421-435) are located within the C-terminal pocket (Supplementary Fig. 14)^15^.

1. **Nsp15 protein**

Nsp15 is a uridylate specific endoribonuclease that is essential to SARS-CoV-2 RNA processing and viral immune evasion ^16^. Nsp15 contains three domains, including the N terminal domain (ND), the middle domain (MD), and a polyU specific endonuclease domain (endoU) with nuclease activity ^17^. In this work, 2 IgM epitopes (nsp15_44-68, nsp15_254-258) and 2 IgG epitopes (nsp15_64-68, nsp15_254-258) were identified (Supplementary Fig. 15a, Supplementary Tables 4 and 5), which are distributed on the protein surface (Supplementary Fig. 15b and 15c).

**References**

1 Wu, Y. *et al.* A noncompeting pair of human neutralizing antibodies block COVID-19 virus binding to its receptor ACE2. *Science* **368**, 1274-1278 (2020).

2 Poh, C. M. *et al.* Two linear epitopes on the SARS-CoV-2 spike protein that elicit neutralising antibodies in COVID-19 patients. *Nat Commun* **11**, 2806 (2020).

3 Wang, H. *et al.* SARS-CoV-2 Proteome Microarray for Mapping COVID-19 Antibody Interactions at Amino Acid Resolution. *ACS Cent Sci* **6**, 2238-2249 (2020).

4 Yoshimoto, F. K. The Proteins of Severe Acute Respiratory Syndrome Coronavirus-2 (SARS CoV-2 or n-COV19), the Cause of COVID-19. *Protein J* **39**, 198-216 (2020).

5 Jiang, H. W. *et al.* SARS-CoV-2 proteome microarray for global profiling of COVID-19 specific IgG and IgM responses. *Nat Commun* **11**, 3581 (2020).

6 Ren, Y. *et al.* The ORF3a protein of SARS-CoV-2 induces apoptosis in cells. *Cell Mol Immunol* **17**, 881-883 (2020).

7 Yuen, C. K. *et al.* SARS-CoV-2 nsp13, nsp14, nsp15 and orf6 function as potent interferon antagonists. *Emerg Microbes Infect* **9**, 1418-1428 (2020).

8 Xia, H. *et al.* Evasion of Type I Interferon by SARS-CoV-2. *Cell Rep* **33**, 108234 (2020).

9 Martin-Sancho, L. *et al.* Functional Landscape of SARS-CoV-2 Cellular Restriction. *bioRxiv* (2020).

10 Flower, T. G. *et al.* Structure of SARS-CoV-2 ORF8, a rapidly evolving coronavirus protein implicated in immune evasion. *bioRxiv preprint doi:* [*https://doi.org/10.1101/2020.08.27.270637*](https://doi.org/10.1101/2020.08.27.270637)*.* (2020).

11 Angeletti, S. *et al.* COVID-2019: The role of the nsp2 and nsp3 in its pathogenesis. *J Med Virol* **92**, 584-588 (2020).

12 Pandey, P., Prasad, K., Prakash, A. & Kumar, V. Insights into the biased activity of dextromethorphan and haloperidol towards SARS-CoV-2 NSP6: in silico binding mechanistic analysis. *J Mol Med (Berl)* **98**, 1659-1673 (2020).

13 Littler, D. R., Gully, B. S., Colson, R. N. & Rossjohn, J. Crystal Structure of the SARS-CoV-2 Non-structural Protein 9, Nsp9. *iScience* **23**, 101258 (2020).

14 Ogando, N. S. *et al.* The Enzymatic Activity of the nsp14 Exoribonuclease Is Critical for Replication of MERS-CoV and SARS-CoV-2. *J Virol* **94**, e01246-01220 (2020).

15 Liu, C. *et al.* Potential Treatment of Chinese and Western Medicine Targeting Nsp14 of SARS-CoV-2. *J Pharm Anal* (2020).

16 Pillon, M. C. *et al.* Cryo-EM Structures of the SARS-CoV-2 Endoribonuclease Nsp15. *bioRxiv* (2020).

17 Kim, Y. *et al.* Crystal structure of Nsp15 endoribonuclease NendoU from SARS-CoV-2. *Protein Sci* **29**, 1596-1605 (2020).

18 Watanabe, Y. *et al.* Vulnerabilities in coronavirus glycan shields despite extensive glycosylation. *Nat Commun* **11**, 2688 (2020).

19 Zhou, D., Tian, X., Qi, R., Peng, C. & Zhang, W. Identification of 22 N-glycosites on spike glycoprotein of SARS-CoV-2 and accessible surface glycopeptide motifs: Implications for vaccination and antibody therapeutics. *Glycobiology* **31**, 69-80 (2021).

**Supplementary Figures.**

**Figure S1. Serum antibody detection workflow using the SARS-CoV-2 proteome microarray.**

**Figure S2 Comparison of the proteome-wide investigations of antibody responses to SARS-CoV-2 infection in this work and previous studies using proteomics technology.** The data set of mild patients was obtained from our previous study by Wang et al. using the same SARS-CoV-2 proteome peptide microarray [PMID: 33372199].


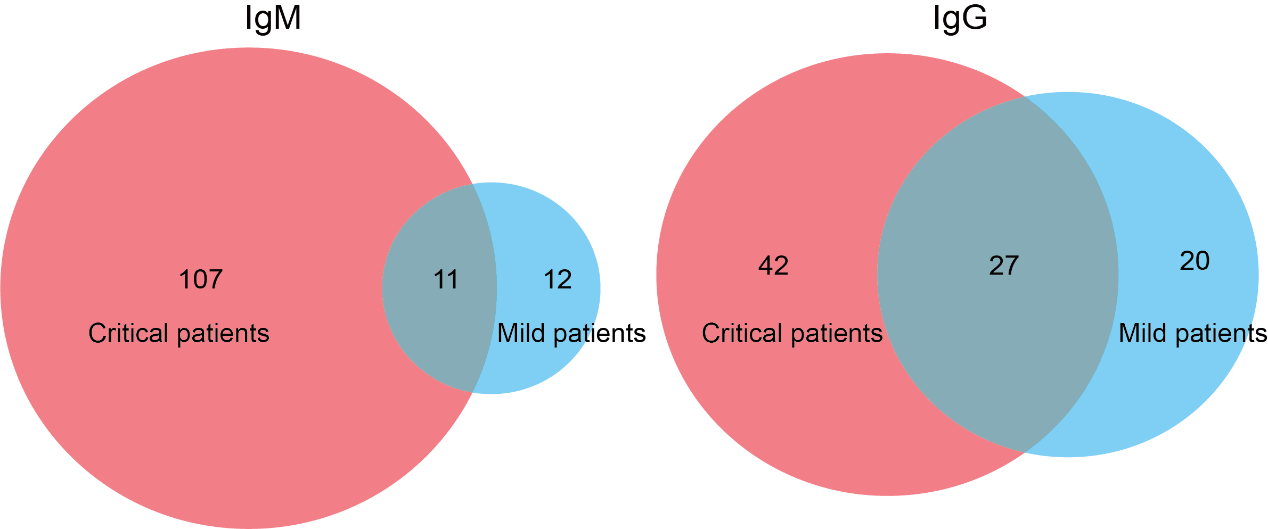


**Figure S3. Comparison of antibody epitopes identified in mild and critical COVID-19 patients. The data set of mild patients was obtained from our previous study using SARS-CoV-2 proteome peptide microarray [PMID: 33372199].**

**
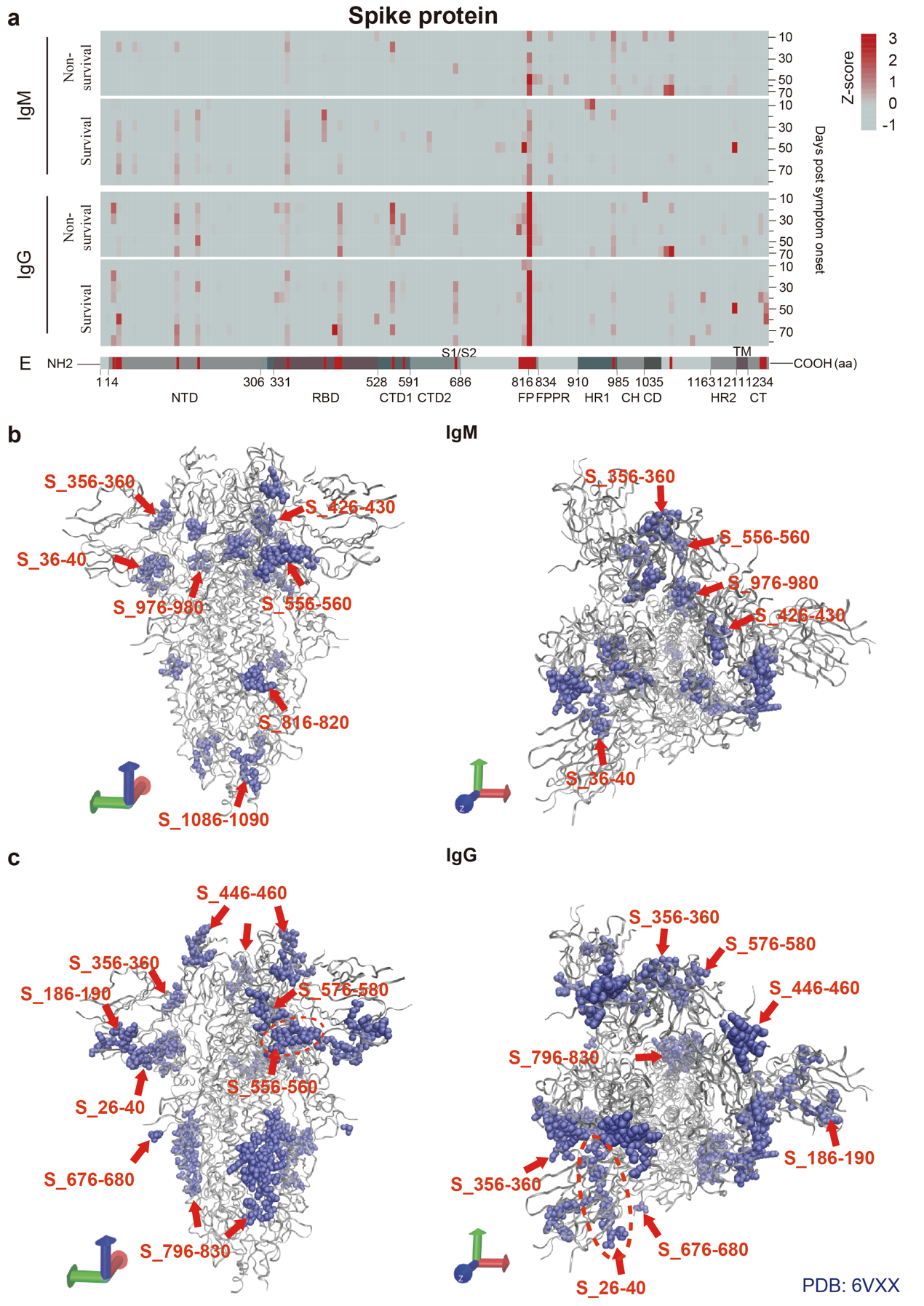
**

**Figure S4. Spatiotemporal resolution analysis of IgM and IgG antibodies to the SARS-CoV-2 Spike protein.** (a) Longitudinal changes of IgM and IgG antibody epitopes within the SARS-CoV-2 S protein. (b, c) Structural analyses of IgM and IgG antibody epitopes within the SARS-CoV-2 S protein (PDB: 6VXX), respectively. The representative epitopes on the protein’s structure are indicated with a red arrow. NTD, N-terminal domain; RBD, receptor binding domain; CTD, C-terminal domain; S2′, S2′ protease cleavage site; FP, fusion peptide; HR1, heptad repeat 1; CH, central helix; CD, connector domain; HR2, heptad repeat 2; TM, transmembrane domain; CT, cytoplasmic tail.

**Figure S5. Structural analysis of the antibody binding epitopes within the S receptor binding domain.** The epitopes are labeled in red.


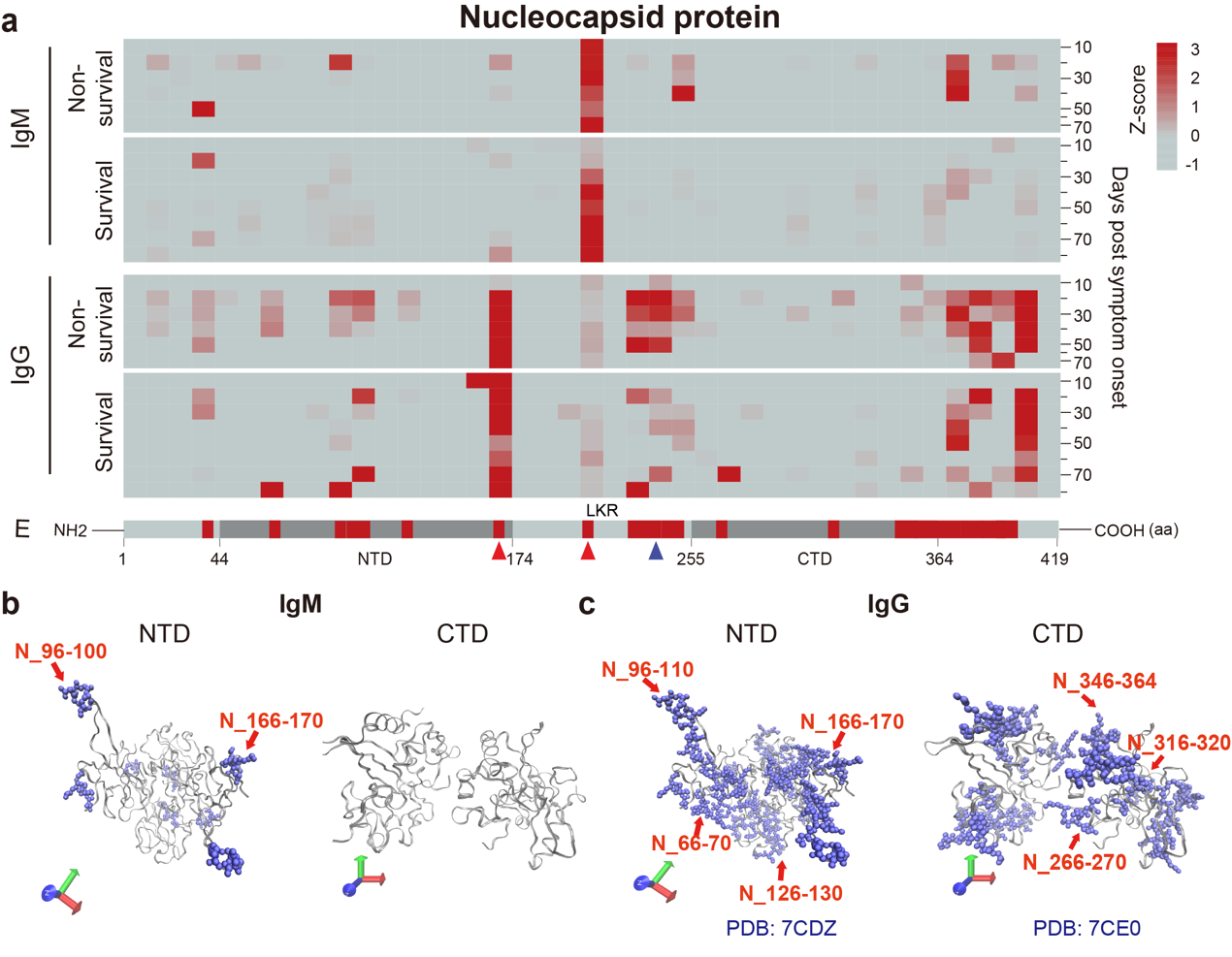


**Figure S6. Spatiotemporal resolution analysis of antibody binding epitopes within the N protein.** (a) Longitudinal changes of IgM and IgG antibody epitopes identified within the SARS-CoV-2 N protein. (b, c) Structural analyses of IgM and IgG antibody epitopes within the SARS-CoV-2 N protein, respectively. The representative epitopes on the protein’s structure are indicated with a red arrow. NTD, N-terminal domain; CTD, C-terminal domain.

**
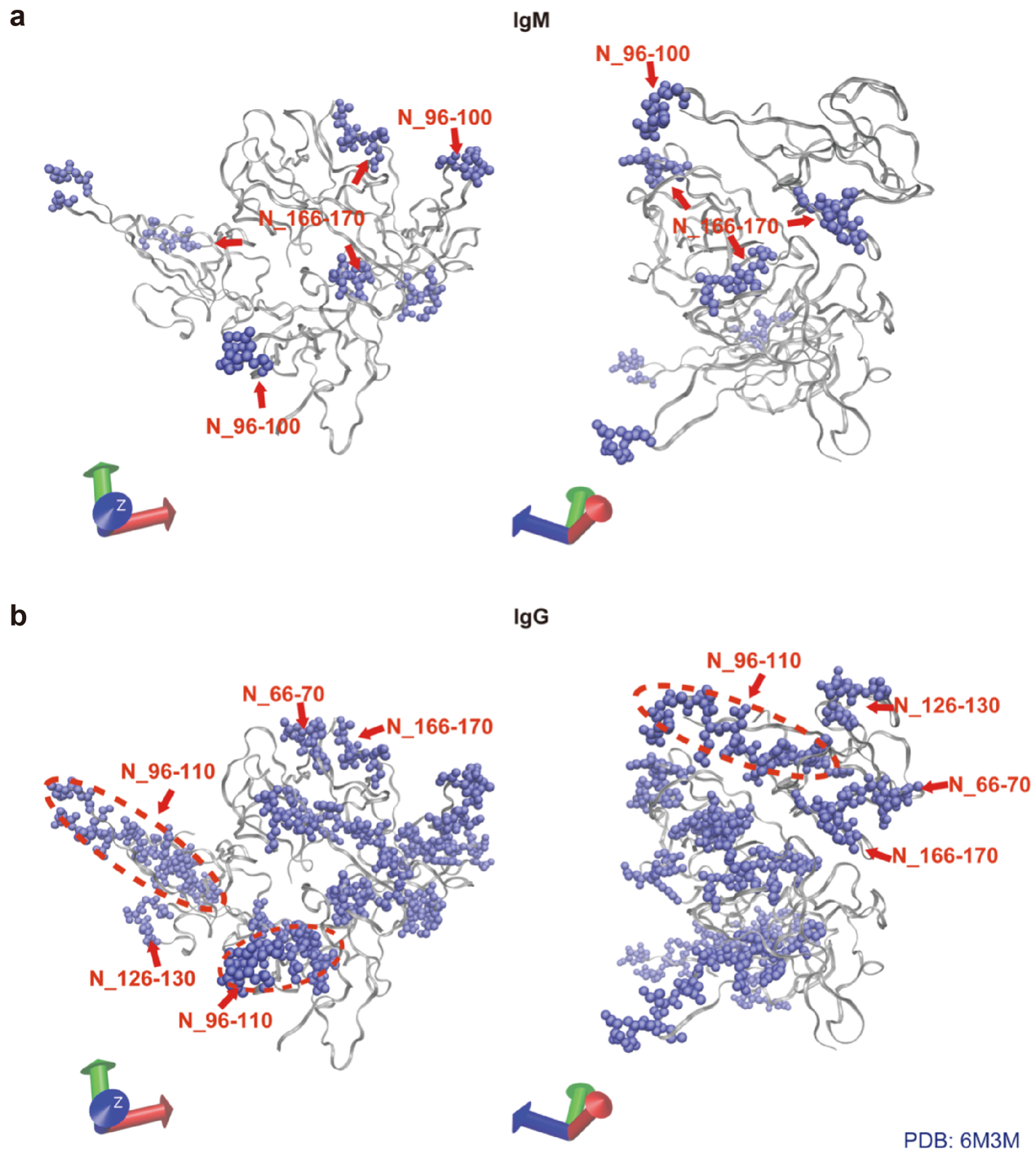
**

**Figure S7. Structural analysis of the antibody binding epitopes within the N protein’s RNA binding domain.** (a) and (b) are the structural analyses of IgM and IgG epitopes, respectively. The epitopes are labeled in red.

**Figure S8. Distribution of antibody epitopes and glycosylation on S the protein.** The glycosylation data was obtained from ^18,19^.


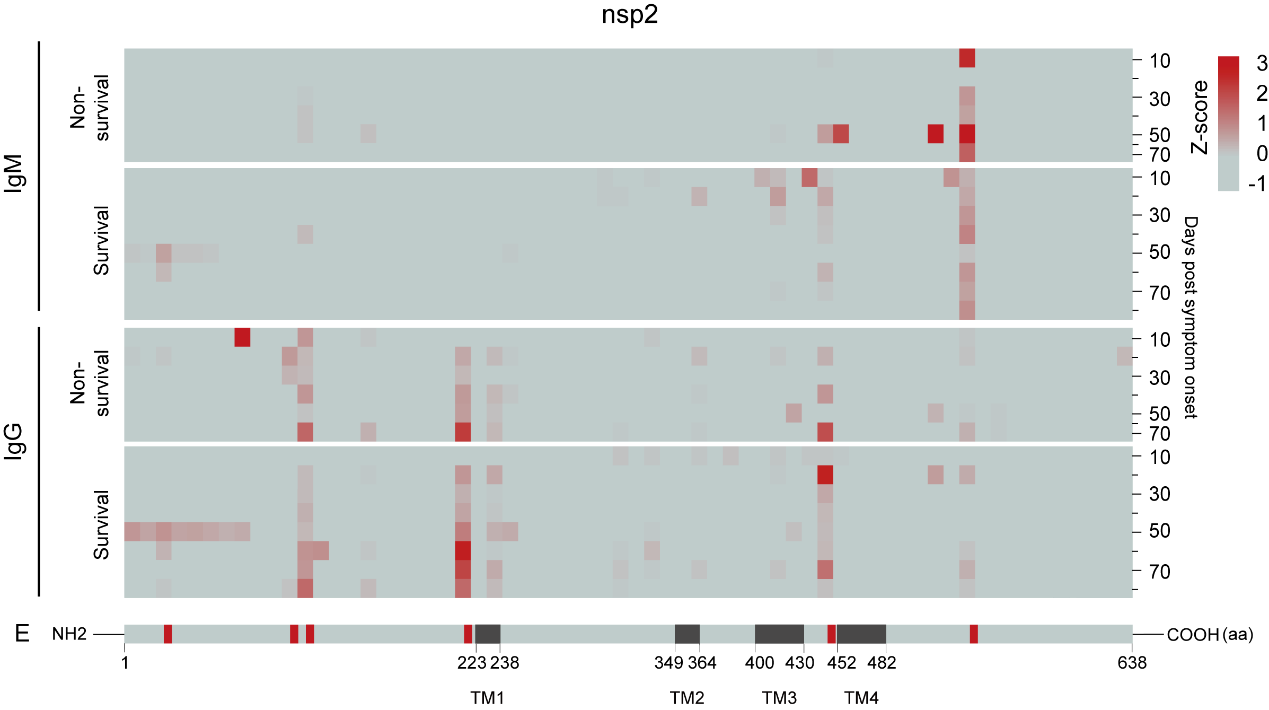


**Figure S9. Longitudinal changes of IgM and IgG antibody binding epitopes with the nsp2 protein.** The epitopes are labeled in red. TM, transmembrane domain.


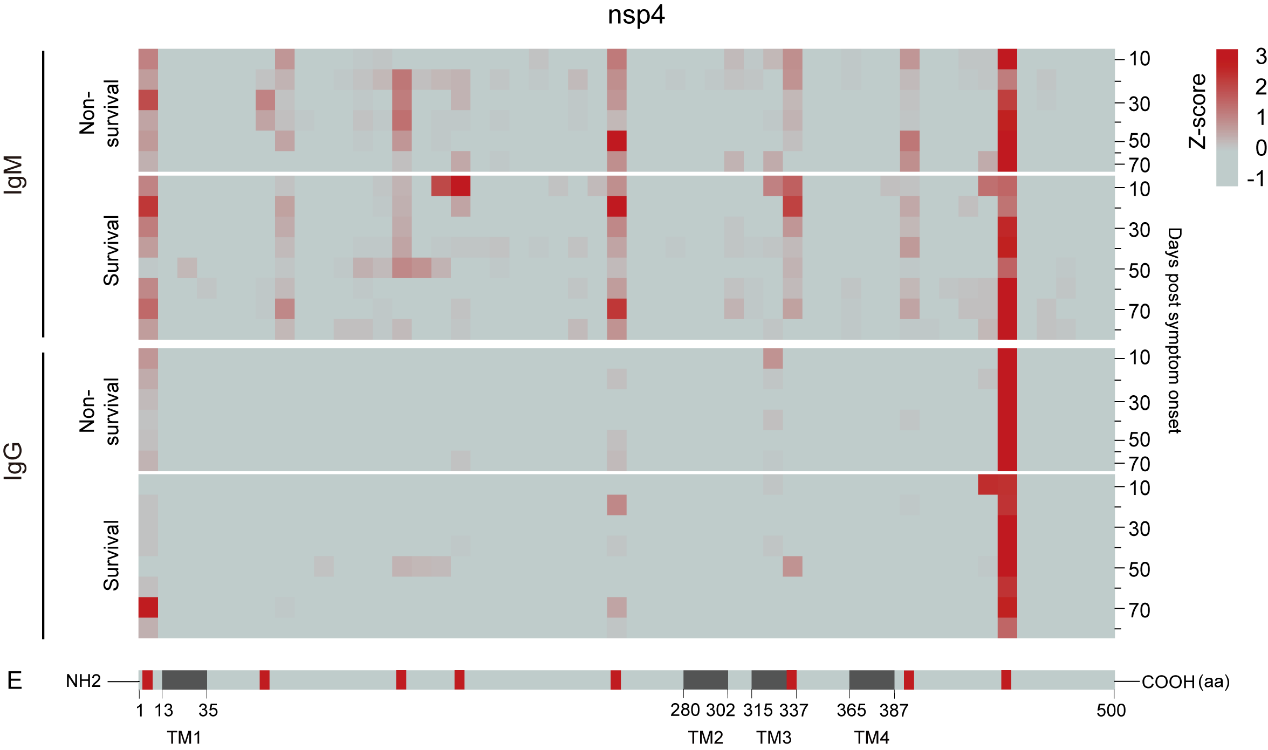


**Figure S10. Longitudinal changes of IgM and IgG antibody binding epitopes with the nsp4 protein.** The epitopes are labeled in red. TM, transmembrane domain.


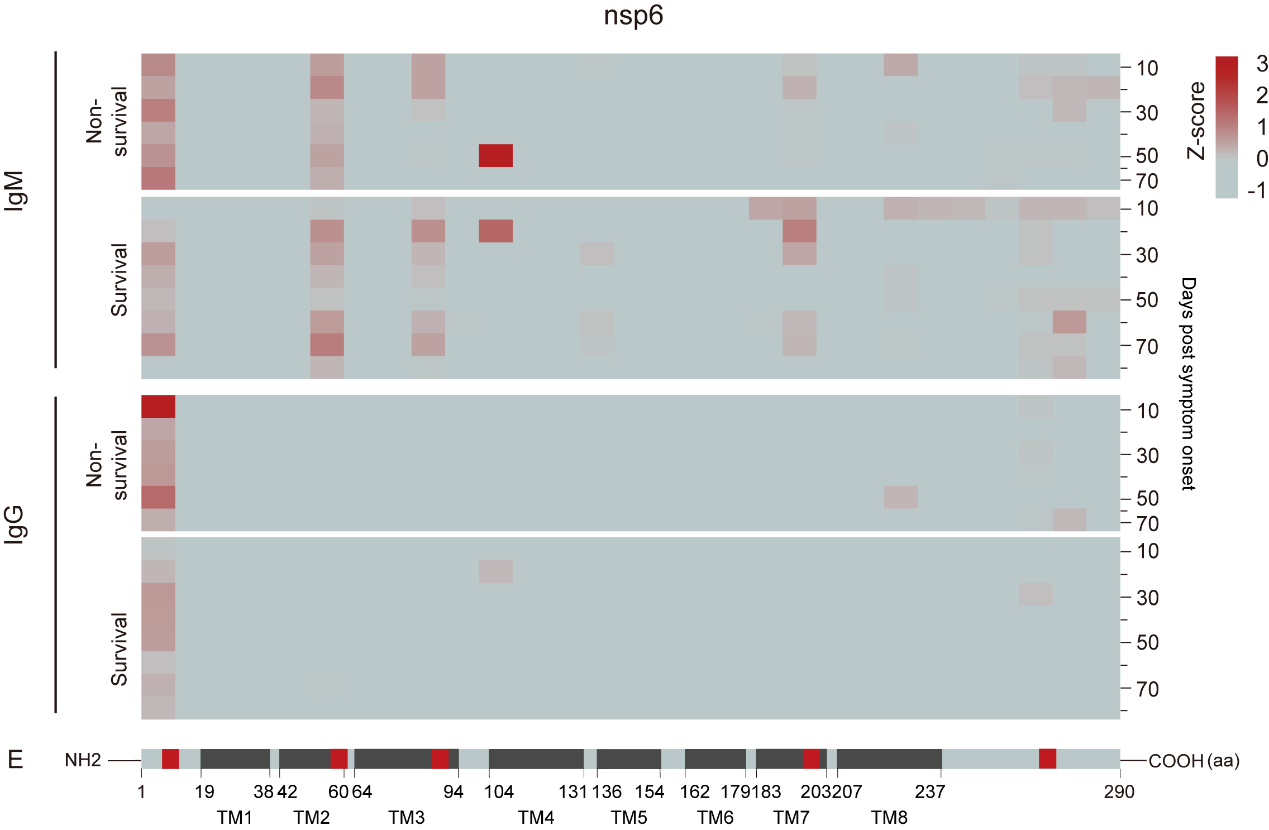


**Figure S11. Longitudinal changes of IgM and IgG antibody binding epitopes with the epitopes on the nsp6 protein.** The epitopes are labeled in red. TM, transmembrane domain.


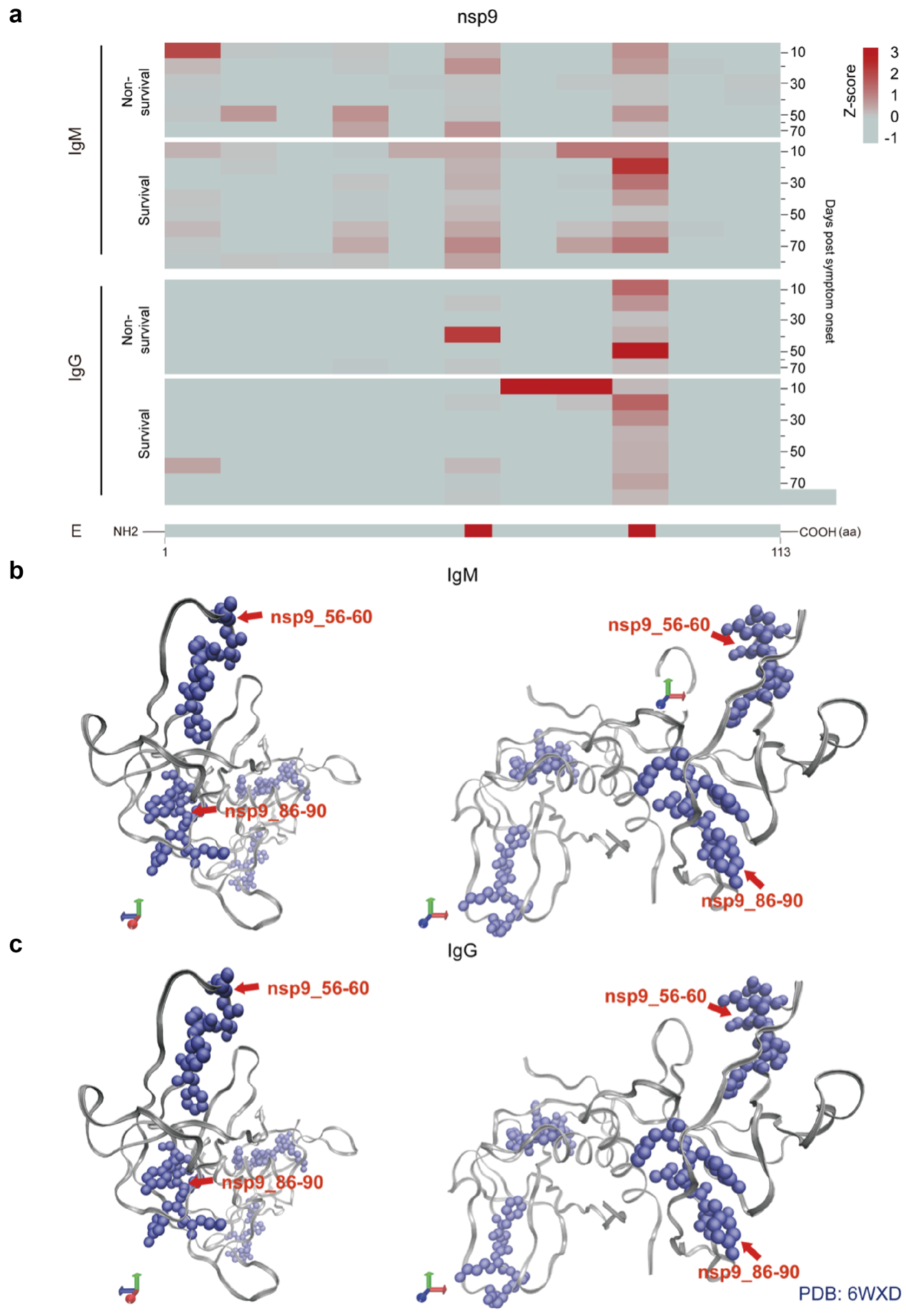


**Figure S12. Spatiotemporal resolution analysis of IgM and IgG antibody binding epitopes with the nsp9 protein.** (a) Longitudinal changes of IgM and IgG antibody epitopes identified within the SARS-CoV-2 nsp9 protein. The epitopes are labeled in red. (b, c) Structural analyses of IgM and IgG epitopes on the SARS-CoV-2 nsp9 protein, respectively.

**
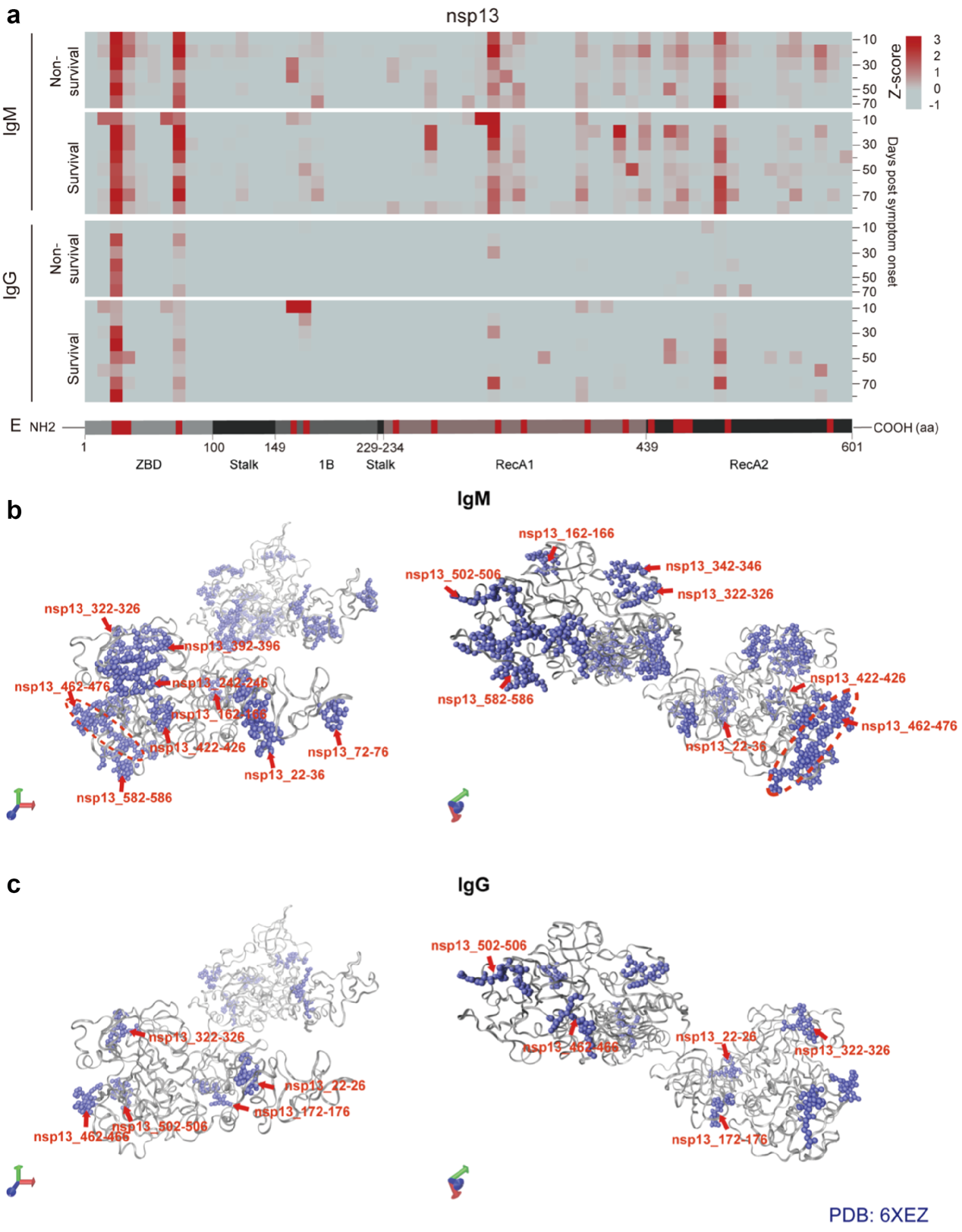
Figure S13. Spatiotemporal resolution analysis of IgM and IgG antibody binding epitopes with the nsp13 protein.** (a) Longitudinal changes of IgM and IgG antibody epitopes identified within the SARS-CoV-2 nsp13 protein. The epitopes are labeled in red. (b, c) Structural analyses of IgM and IgG epitopes on the SARS-CoV-2 nsp13 protein, respectively. ZBD, zinc-binding domain.


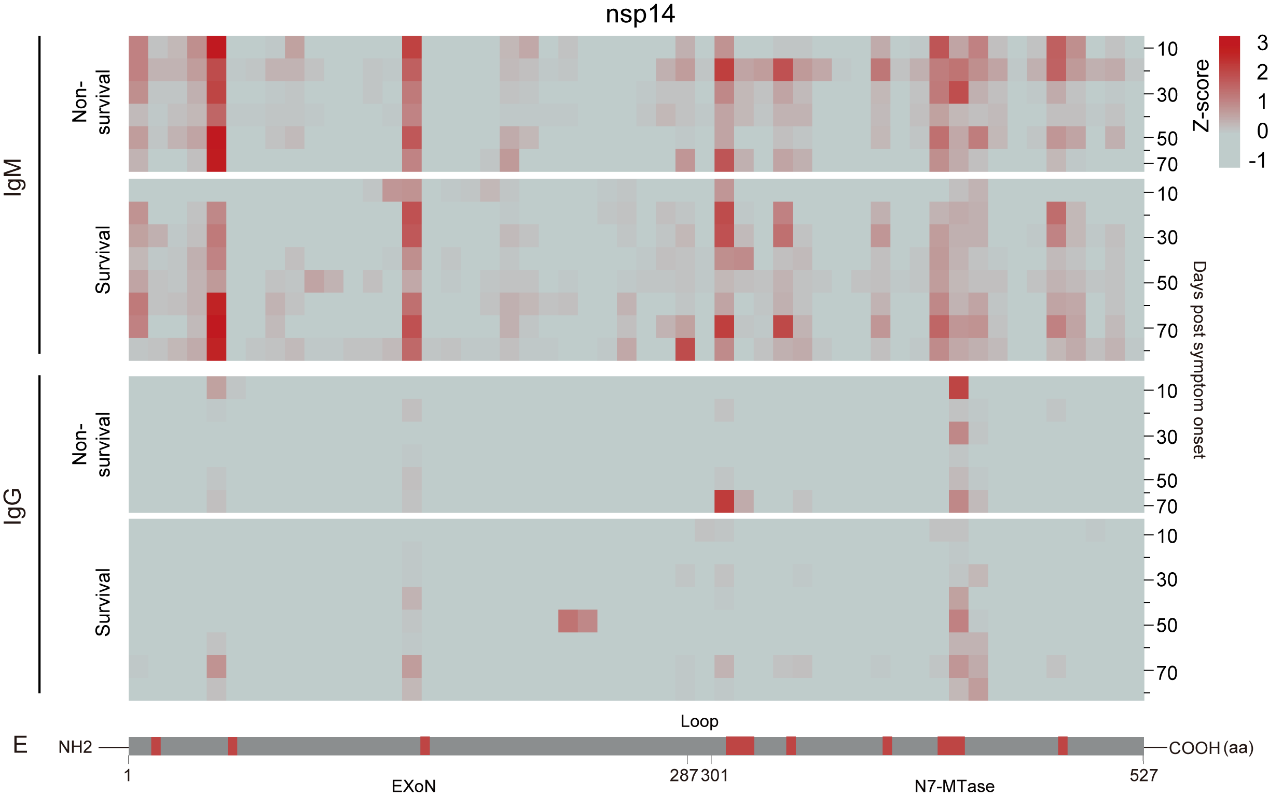


**Figure S14. Longitudinal changes of IgM and IgG antibody binding epitopes with the nsp14 protein.** The epitopes are labeled in red. EXoN, 3′-5′ exoribonuclease; N7-MTase, N7-methyl transferase.


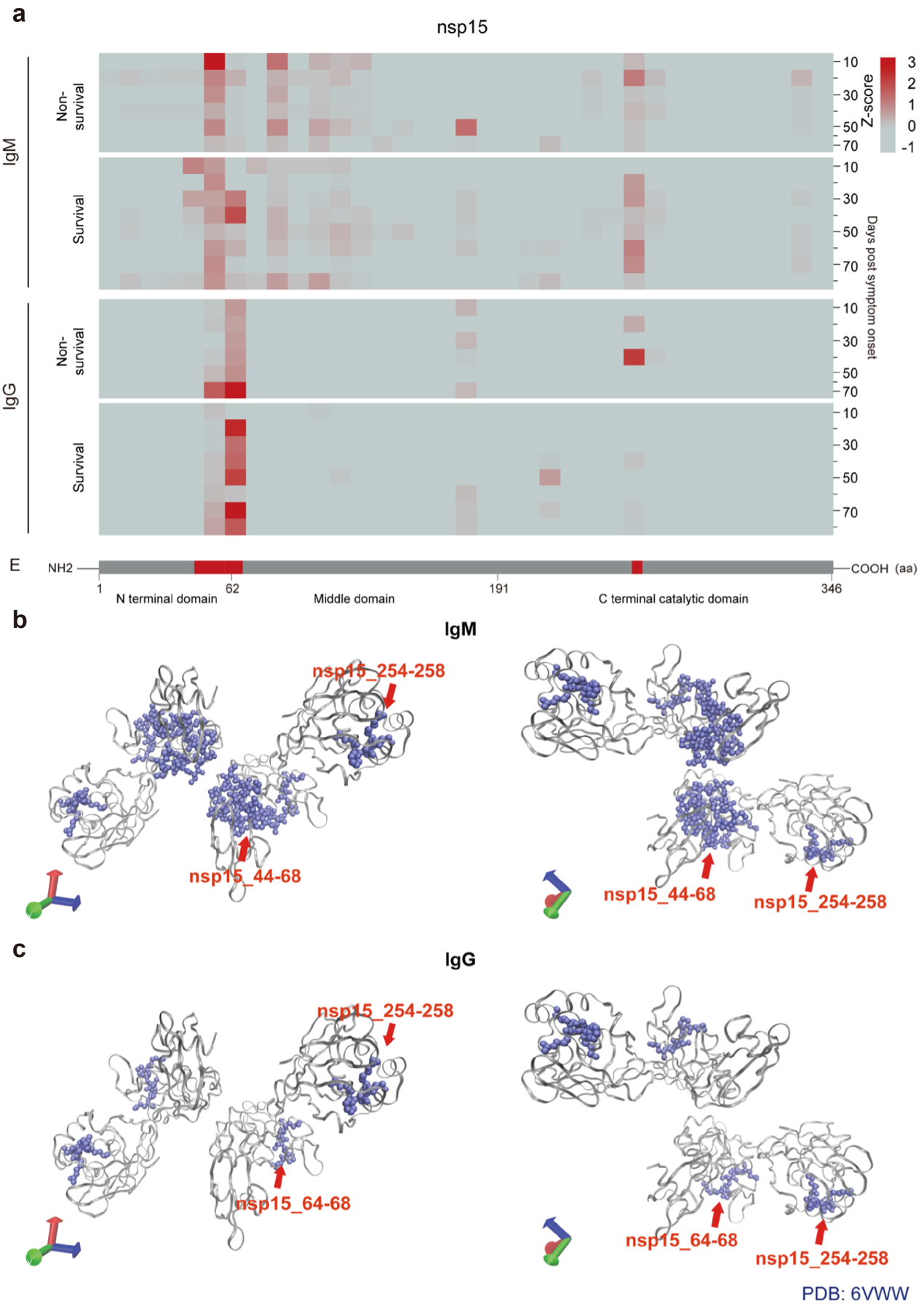


**Figure S15. Spatiotemporal resolution analysis of IgM and IgG antibody binding epitopes with the nsp15 protein.** (a) Longitudinal changes of IgM and IgG antibody epitopes identified within the SARS-CoV-2 nsp15 protein. The epitopes are labeled in red. (b, c) Structural analyses of IgM and IgG epitopes on the SARS-CoV-2 nsp15 protein, respectively.


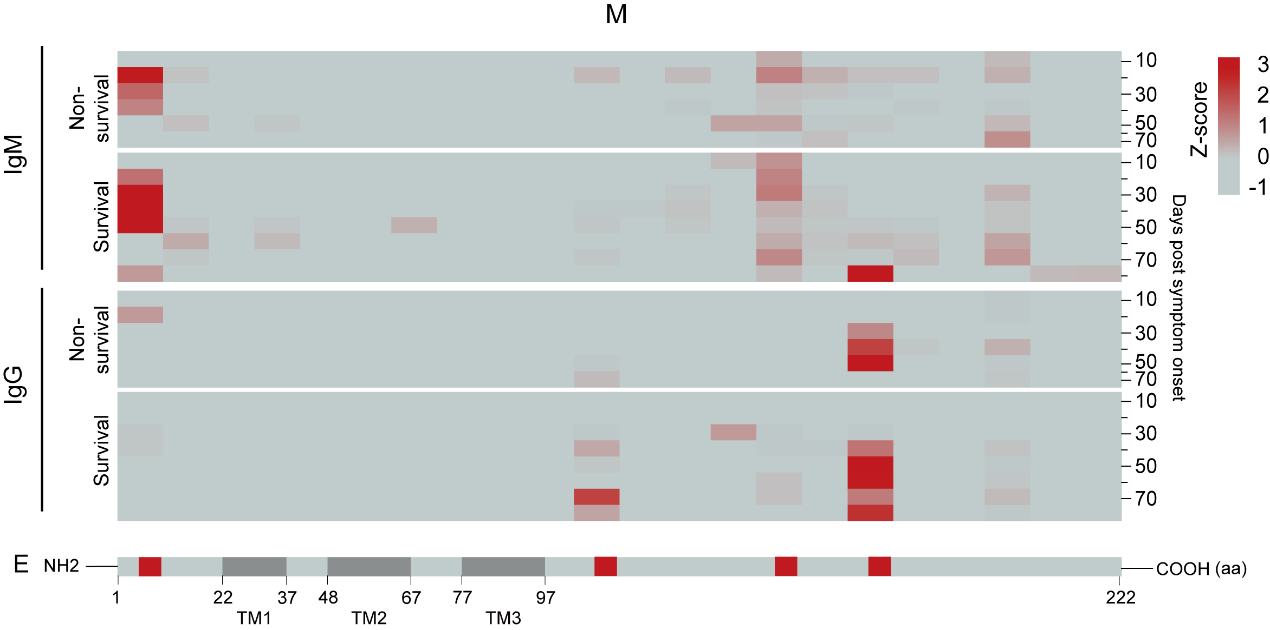


**Figure S16. Longitudinal changes of IgM and IgG antibody binding epitopes with the M protein.** The epitopes on the protein sequence is labeled in red. TM, transmembrane domain.

**
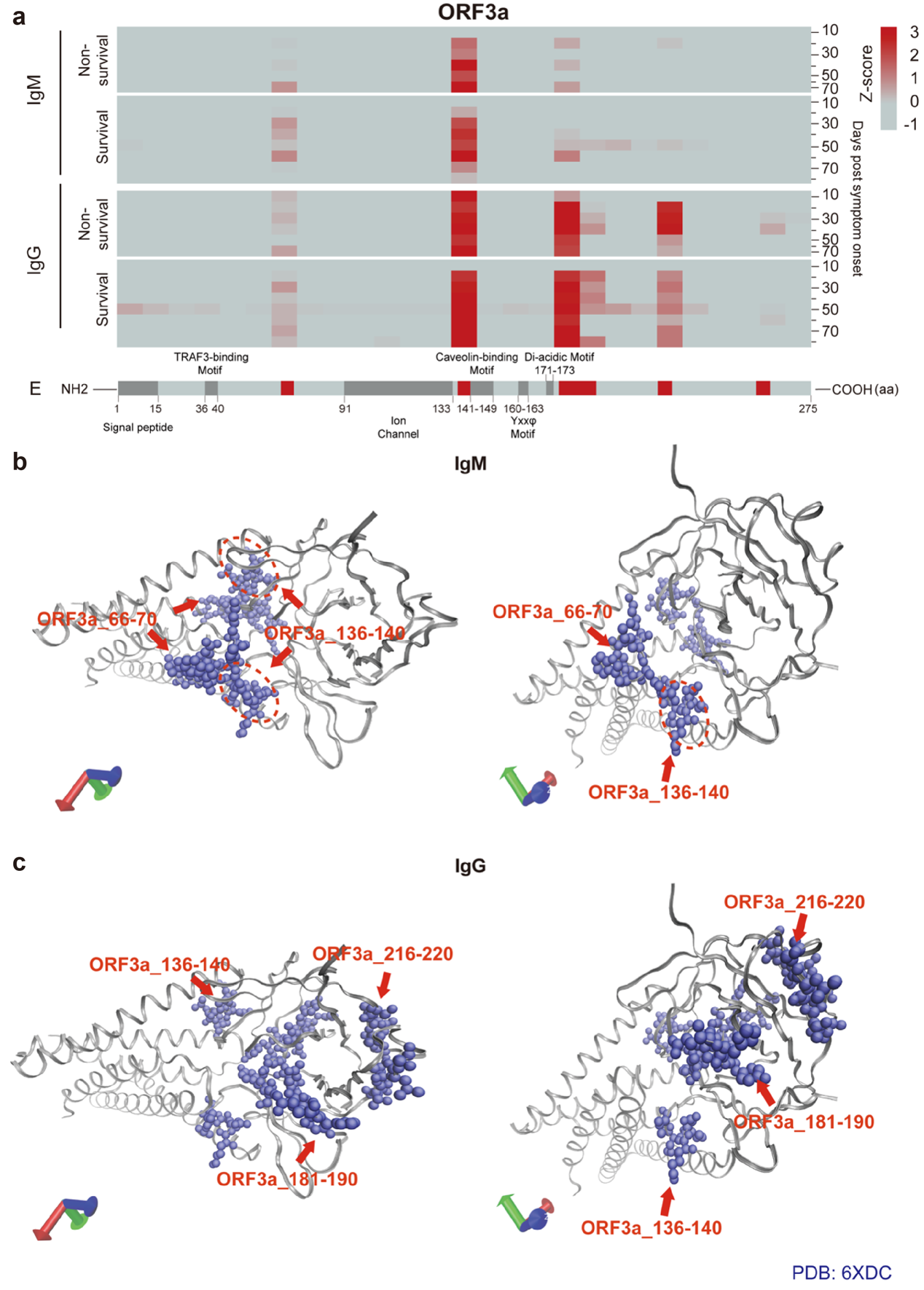
**

**Figure S17.** **Spatiotemporal resolution analysis of IgM and IgG antibody binding epitopes with the ORF3a protein.** (a) Longitudinal changes of IgM and IgG antibody epitopes identified within the SARS-CoV-2 ORF3a protein. The epitopes are labeled in red. (b, c) Structural analyses of IgM and IgG epitopes within the SARS-CoV-2 ORF3a protein, respectively.


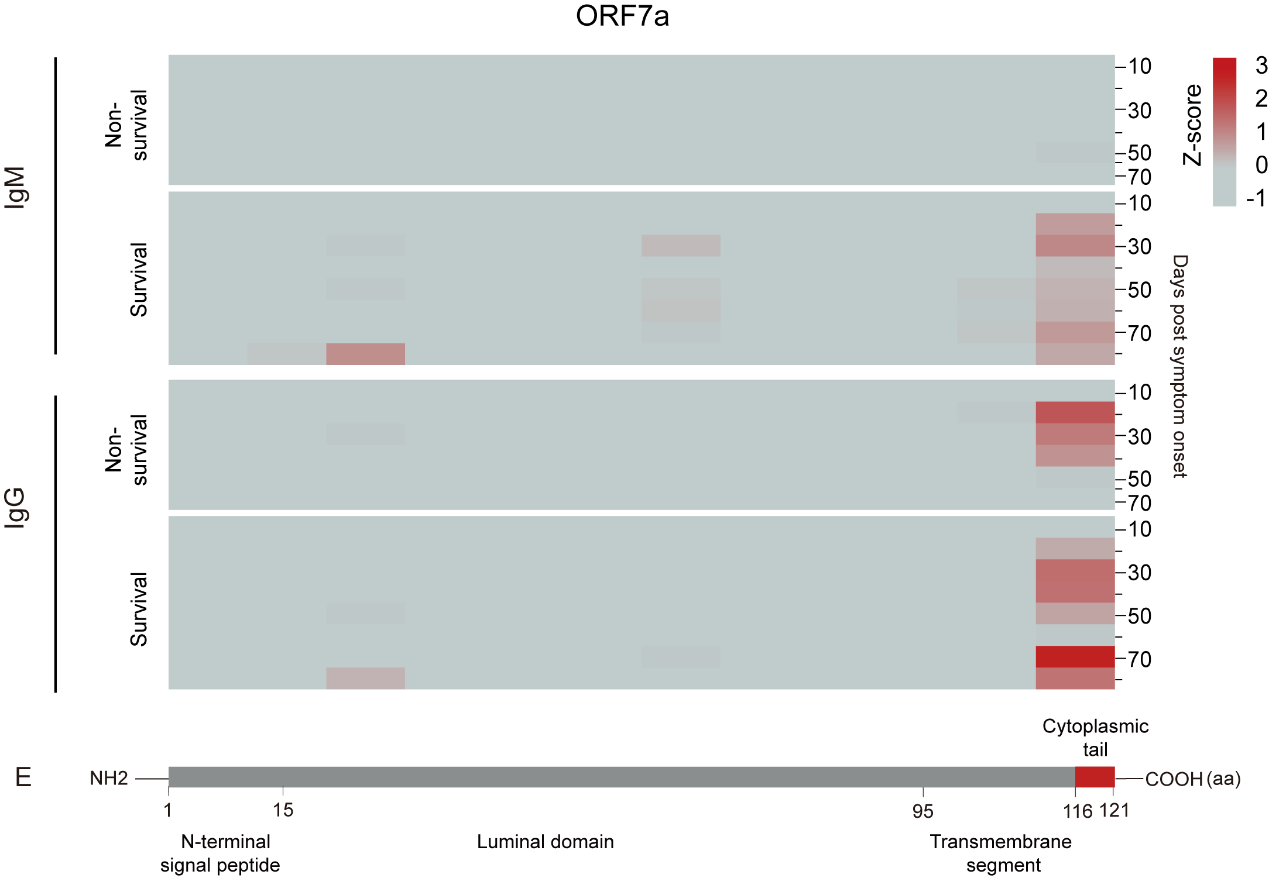


**Figure S18. Longitudinal changes of IgM and IgG antibody binding epitopes with the ORF7a protein.** The epitopes are labeled in red.


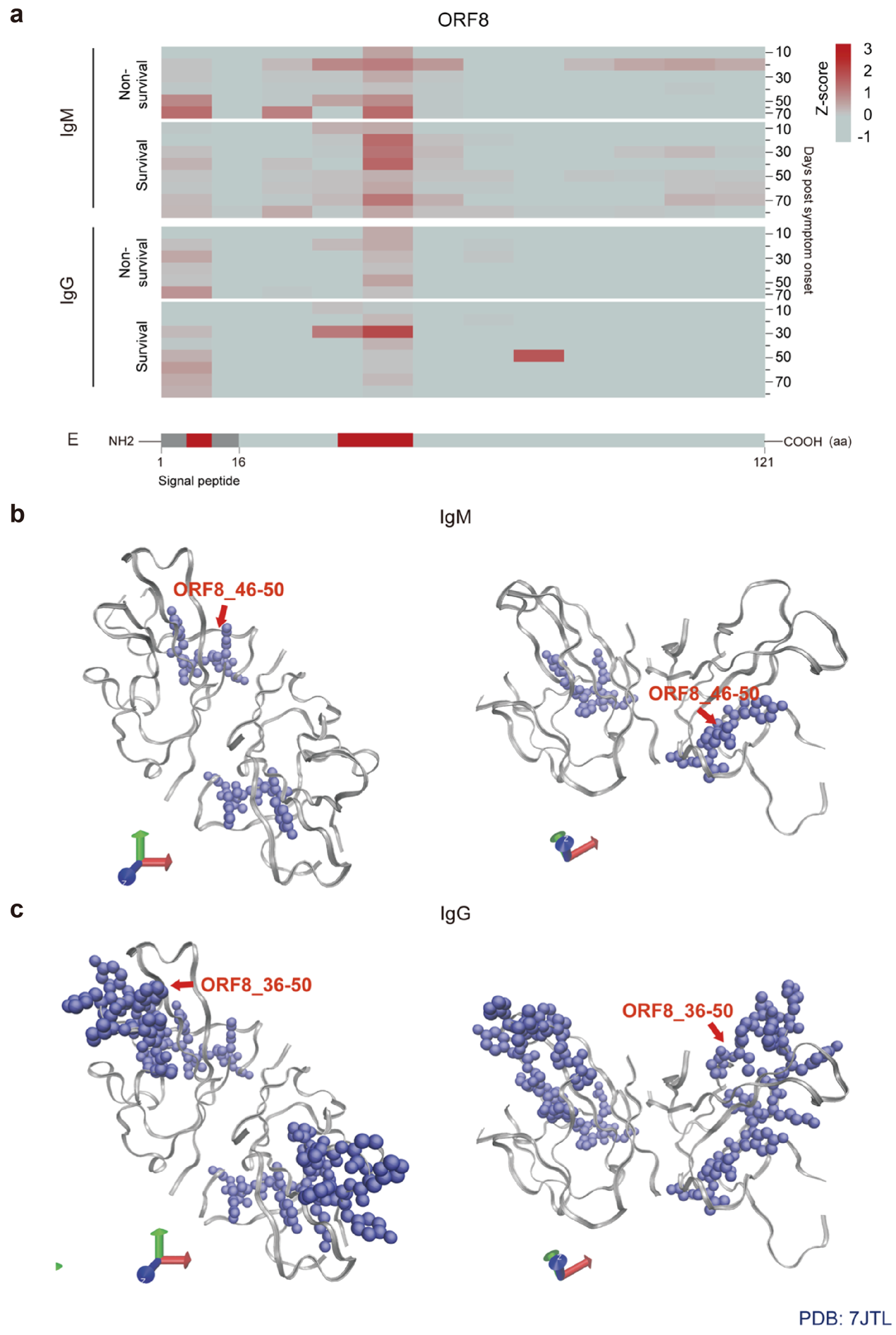


**Figure S19. Spatiotemporal resolution analysis of IgM and IgG antibody binding epitopes with the ORF8 protein.** (a) Longitudinal changes of IgM and IgG antibody epitopes identified within the SARS-CoV-2 ORF8 protein. The epitopes are labeled in red. (b, c) Structural analyses of IgM and IgG epitopes within the SARS-CoV-2 ORF8 protein, respectively.


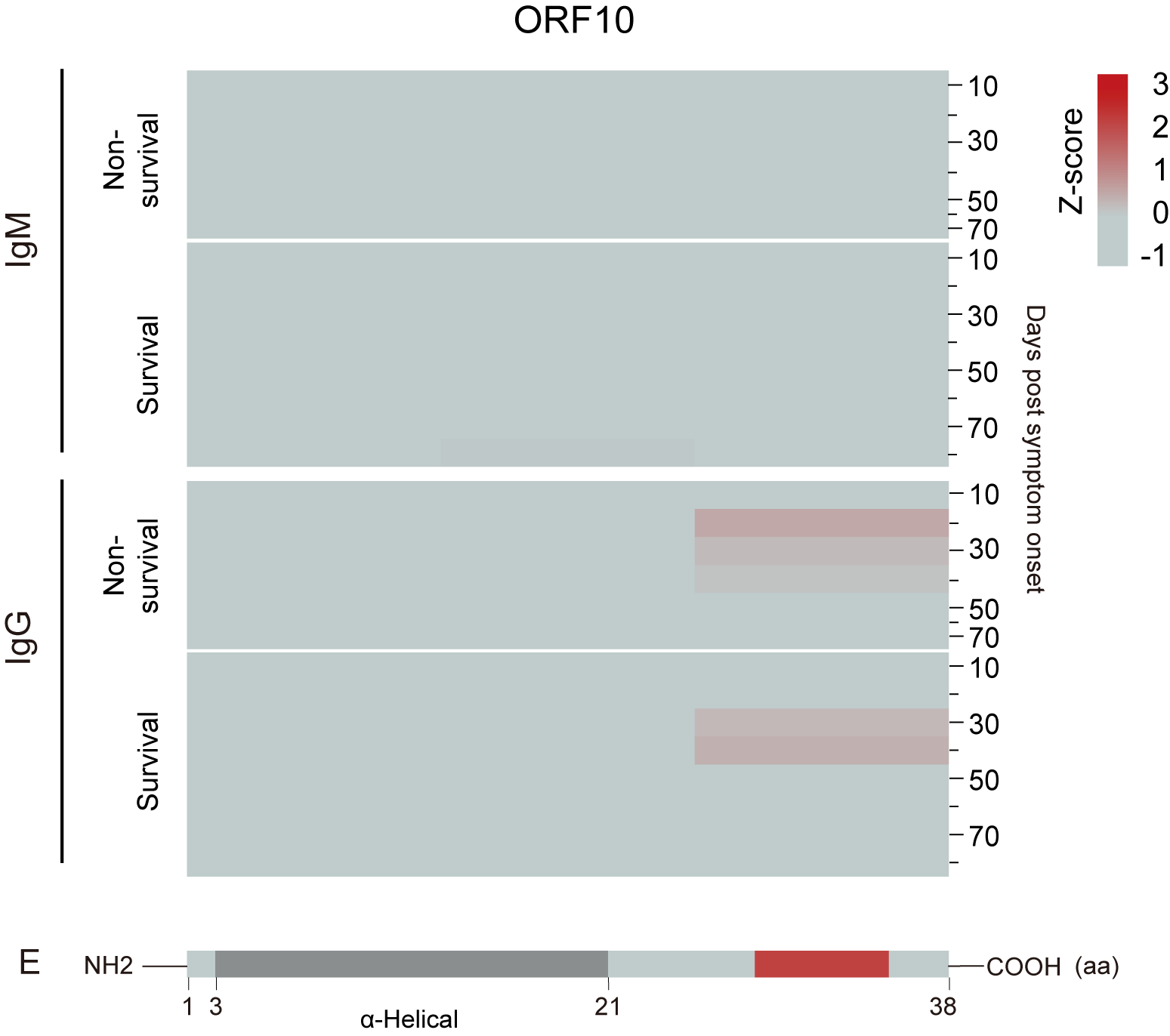


**Figure S20. Longitudinal changes of IgM and IgG antibody binding epitopes with the ORF10 protein.** The epitopes are labeled in red.

**
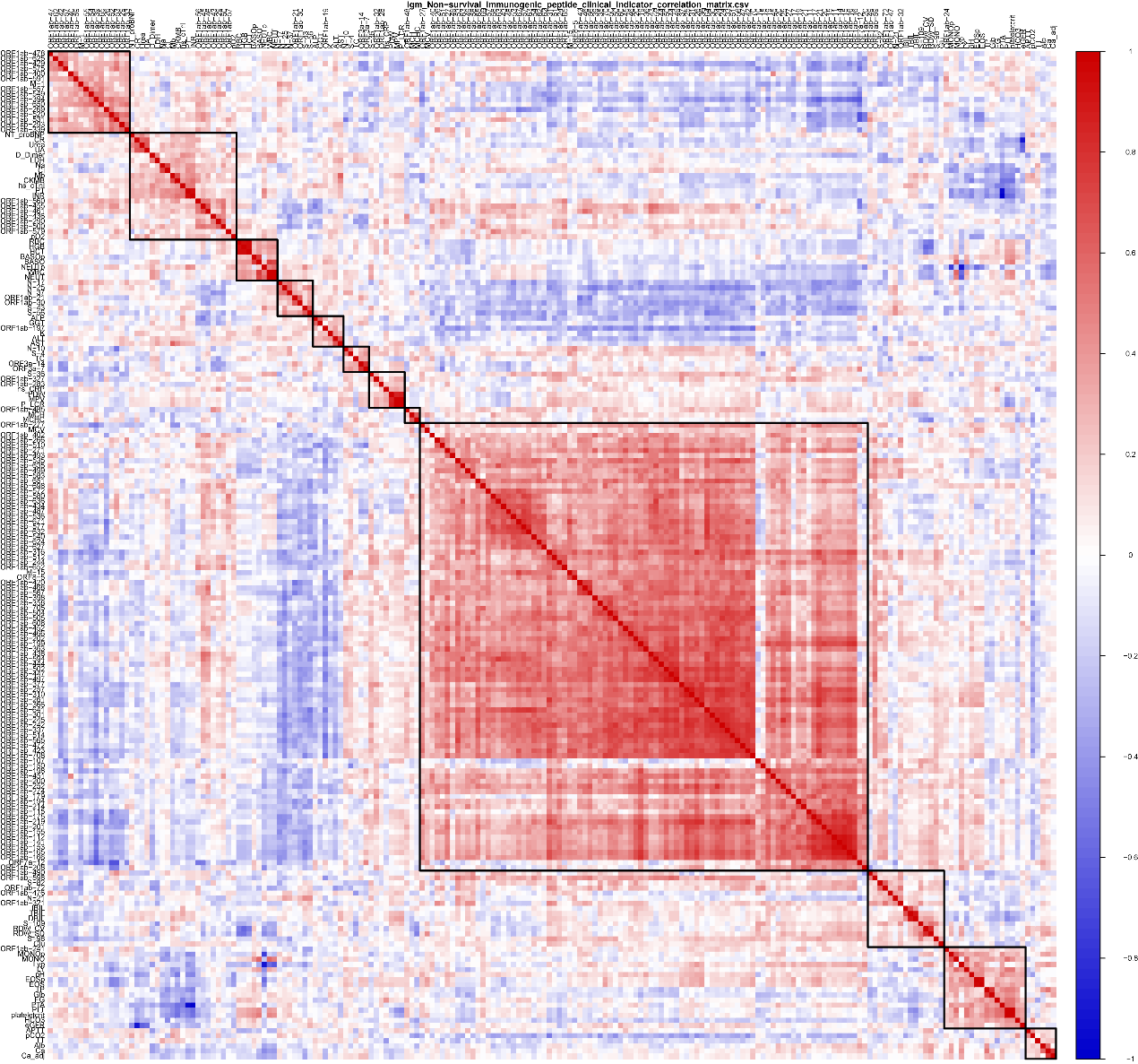
**

**Figure S21. Global correlation map of the SARS-CoV-2 proteome IgM antibodies and clinical variables in the non-survival COVID-19 patient group.** The rainbow color from blue to red corresponds to the correlation of two variables from -1 (low correlation; blue) to +1 (high correlation; red).

**
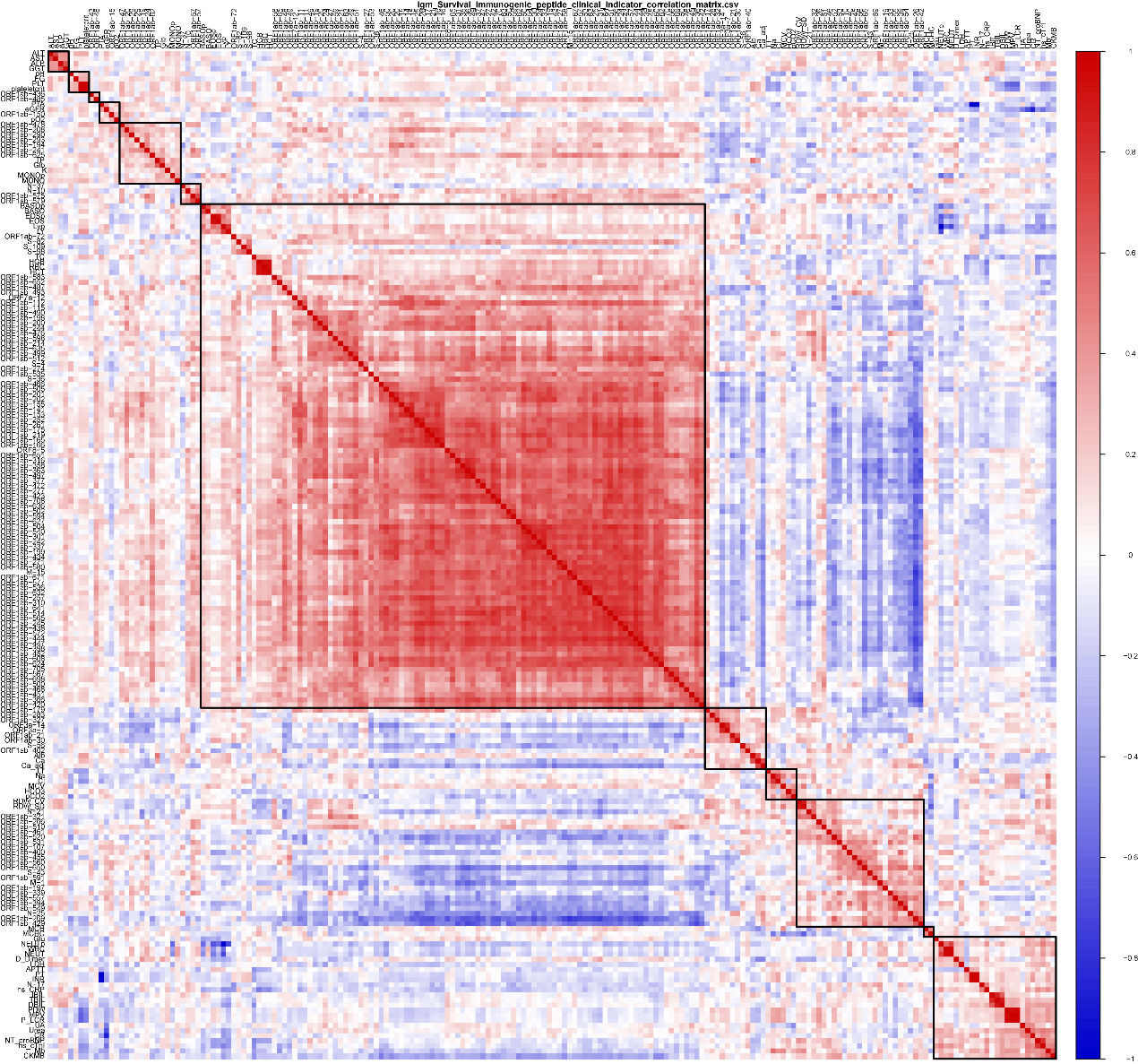
**

**Figure S22. Global correlation map of the SARS-CoV-2 proteome IgM antibodies and clinical variables in the survival COVID-19 patient group.** The rainbow color from blue to red corresponds to the correlation of two variables from -1 (low correlation; blue) to +1 (high correlation; red).

**
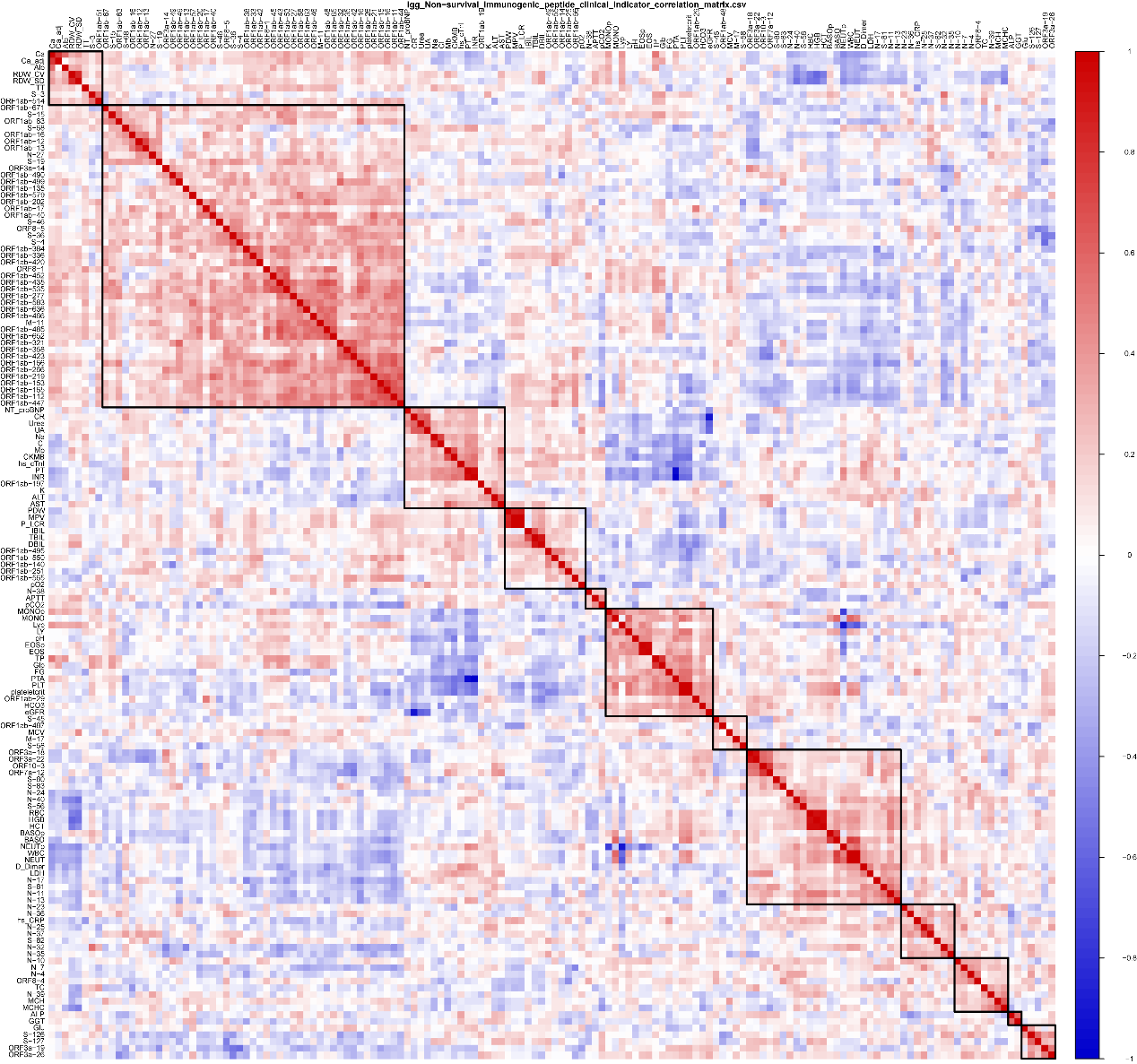
**

**Figure S23. Global correlation map of the SARS-CoV-2 proteome IgG antibodies and clinical variables in the non-survival COVID-19 patient group.** The rainbow color from blue to red corresponds to the correlation of two variables from -1 (low correlation; blue) to +1 (high correlation; red).

**
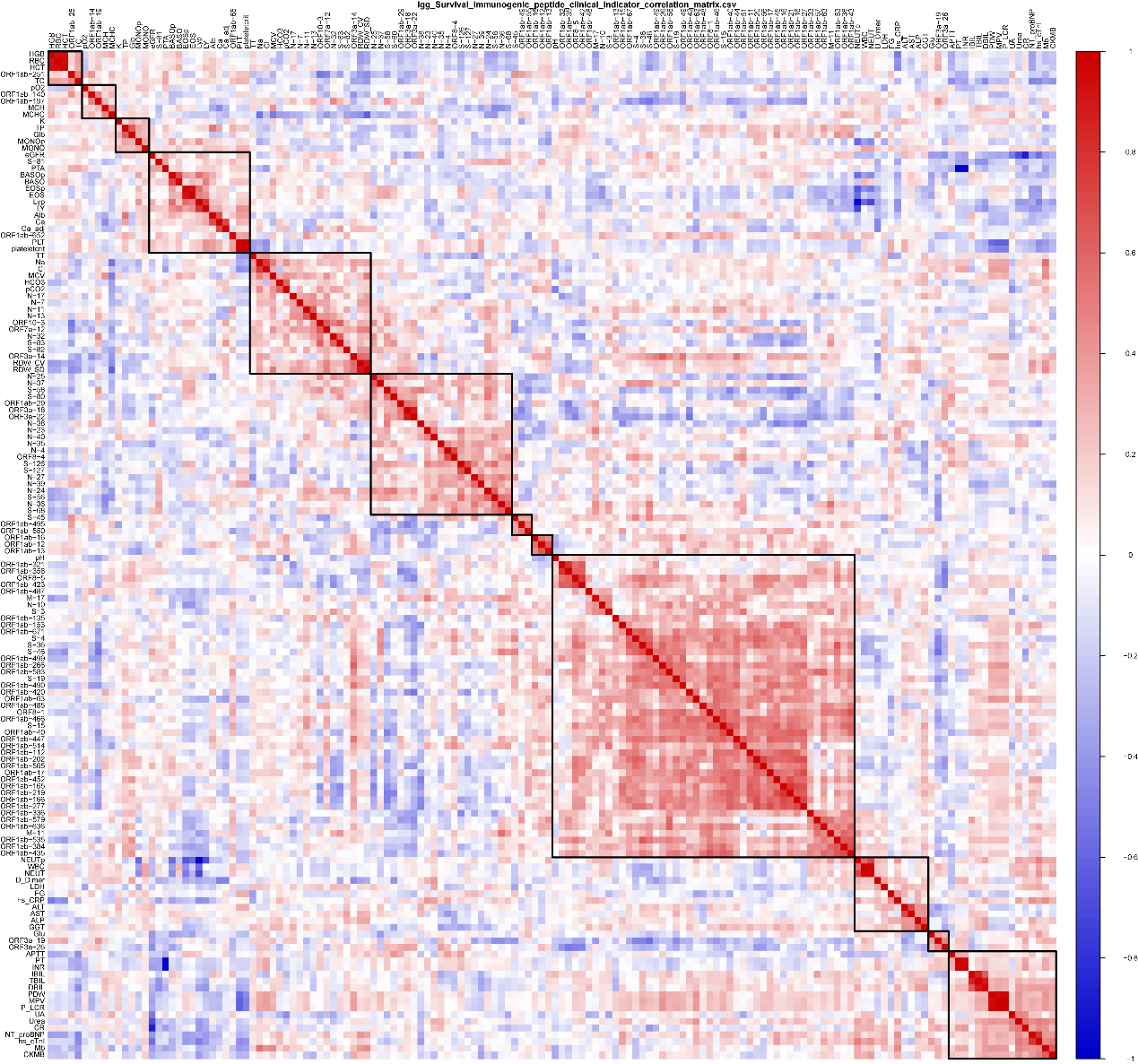
**

**Figure S24. Global correlation map of the SARS-CoV-2 proteome IgG antibodies and clinical variables in the survival COVID-19 patient group.** The rainbow color from blue to red corresponds to the correlation of two variables from -1 (low correlation; blue) to +1 (high correlation; red).

**
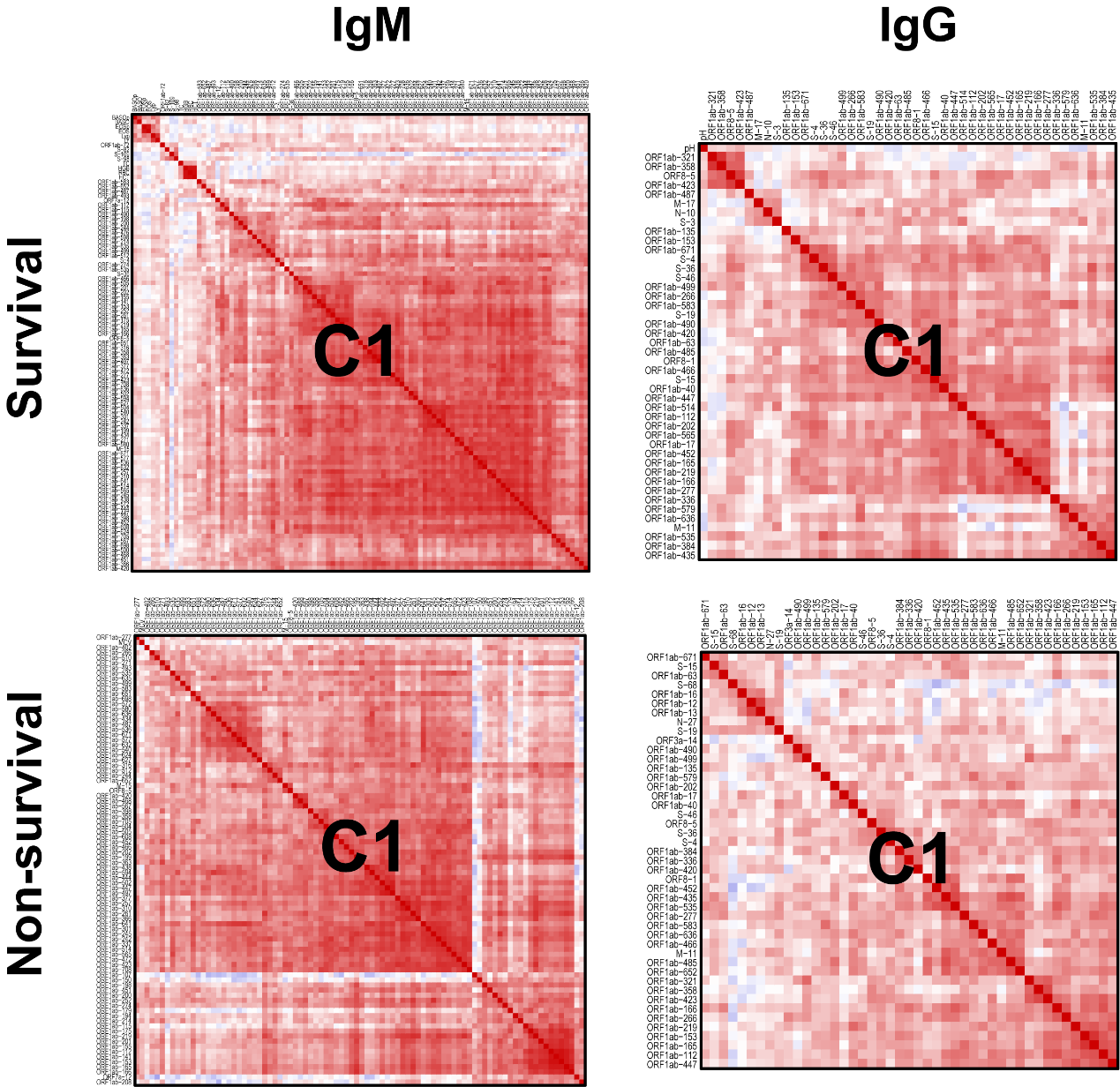
**

**Figure S25. SARS-CoV-2 antibodies in the cluster #1 group.** The rainbow color from blue to red corresponds to the correlation of two variables from -1 (low correlation; blue) to +1 (high correlation; red).


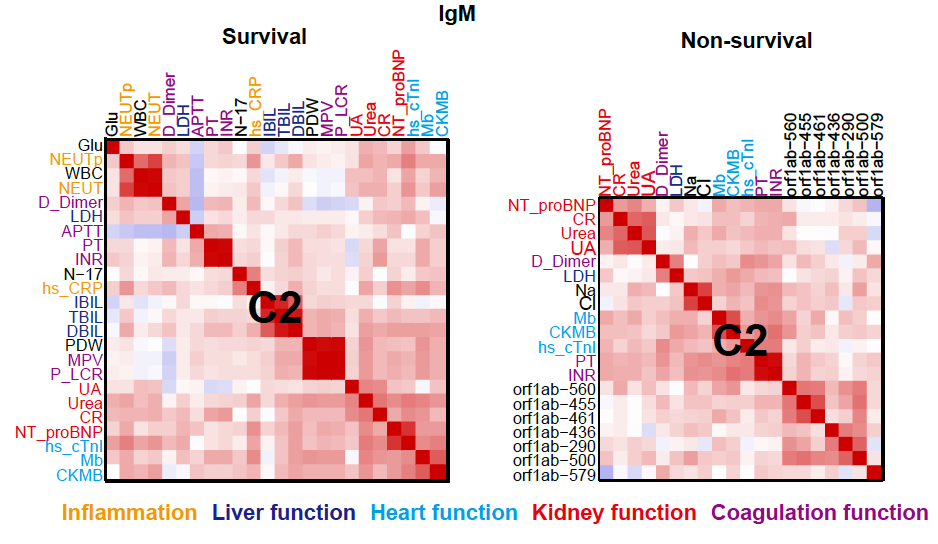


**Figure S26. Differential correlations of the clinical variables and SARS-CoV-2 IgM antibodies between survival and non-survival COVID-19 groups in the cluster #2 group.** The rainbow color from blue to red corresponds to the correlation of two variables from -1 (low correlation; blue) to +1 (high correlation; red).
